# Air Pollution Exposure and Lung Function in Children: The ESCAPE Project

**DOI:** 10.1289/ehp.1306770

**Published:** 2013-09-27

**Authors:** Ulrike Gehring, Olena Gruzieva, Raymond M. Agius, Rob Beelen, Adnan Custovic, Josef Cyrys, Marloes Eeftens, Claudia Flexeder, Elaine Fuertes, Joachim Heinrich, Barbara Hoffmann, Johan C. de Jongste, Marjan Kerkhof, Claudia Klümper, Michal Korek, Anna Mölter, Erica S. Schultz, Angela Simpson, Dorothea Sugiri, Magnus Svartengren, Andrea von Berg, Alet H. Wijga, Göran Pershagen, Bert Brunekreef

**Affiliations:** 1 Institute for Risk Assessment Sciences, Utrecht University, Utrecht, the Netherlands; 2 Institute of Environmental Medicine, Karolinska Institutet, Stockholm, Sweden; 3 Centre for Epidemiology, Institute of Population Health, Manchester Academic Health Sciences Centre, The University of Manchester, Manchester, United Kingdom; 4 Institute of Inflammation and Repair, Manchester Academic Health Science Centre, The University of Manchester and University Hospital of South Manchester NHS Foundation Trust, Manchester, United Kingdom; 5 Institute of Epidemiology II, Helmholtz Zentrum, München, German Research Centre for Environmental Health, Neuherberg, Germany; 6 Environment Science Center, The University of Augsburg, Augsburg, Germany; 7 Institute of Epidemiology I, Helmholtz Zentrum, München, German Research Centre for Environmental Health, Neuherberg, Germany; 8 IUF-Leibniz Research Institute for Environmental Medicine at the University of Düsseldorf, Düsseldorf, Germany; 9 Medical Faculty, Heinrich-Heine-University Düsseldorf, Düsseldorf, Germany; 10 Department of Pediatrics, Division of Respiratory Medicine, Erasmus University Medical Center/Sophia Children’s Hospital, Rotterdam, the Netherlands; 11 Department of Epidemiology, University Medical Center Groningen, University of Groningen, Groningen, the Netherlands; 12 Department of Medical Sciences, Uppsala University, Uppsala, Sweden; 13 Department of Pediatrics, Research Institute, Marien Hospital Wesel, Wesel, Germany; 14 Center for Nutrition, Prevention and Health Services, National Institute of Public Health and the Environment, Bilthoven, the Netherlands; 15 Julius Center for Health Sciences and Primary Care, University Medical Center Utrecht, Utrecht, the Netherlands

## Abstract

Background: There is evidence for adverse effects of outdoor air pollution on lung function of children. Quantitative summaries of the effects of air pollution on lung function, however, are lacking due to large differences among studies.

Objectives: We aimed to study the association between residential exposure to air pollution and lung function in five European birth cohorts with a standardized exposure assessment following a common protocol.

Methods: As part of the European Study of Cohorts for Air Pollution Effects (ESCAPE) we analyzed data from birth cohort studies situated in Germany, Sweden, the Netherlands, and the United Kingdom that measured lung function at 6–8 years of age (*n* = 5,921). Annual average exposure to air pollution [nitrogen oxides (NO_2_, NO_x_), mass concentrations of particulate matter with diameters < 2.5, < 10, and 2.5–10 μm (PM_2.5_, PM_10_, and PM_coarse_), and PM_2.5_ absorbance] at the birth address and current address was estimated by land-use regression models. Associations of lung function with estimated air pollution levels and traffic indicators were estimated for each cohort using linear regression analysis, and then combined by random effects meta-analysis.

Results: Estimated levels of NO_2_, NO_x_, PM_2.5_ absorbance, and PM_2.5_ at the current address, but not at the birth address, were associated with small decreases in lung function. For example, changes in forced expiratory volume in 1 sec (FEV_1_) ranged from –0.86% (95% CI: –1.48, –0.24%) for a 20-μg/m^3^ increase in NO_x_ to –1.77% (95% CI: –3.34, –0.18%) for a 5-μg/m^3^ increase in PM_2.5_.

Conclusions: Exposure to air pollution may result in reduced lung function in schoolchildren.

Citation: Gehring U, Gruzieva O, Agius RM, Beelen R, Custovic A, Cyrys J, Eeftens M, Flexeder C, Fuertes E, Heinrich J, Hoffmann B, de Jongste JC, Kerkhof M, Klümper C, Korek M, Mölter A, Schultz ES, Simpson A, Sugiri D, Svartengren M, von Berg A, Wijga AH, Pershagen G, Brunekreef B. 2013. Air pollution exposure and lung function in children: the ESCAPE project. Environ Health Perspect 121:1357–1364; http://dx.doi.org/10.1289/ehp.1306770

## Introduction

Lung function is an objective marker of respiratory health and a predictor of cardiorespiratory morbidity and mortality (Sin et al. 2005). The long-term effects of ambient air pollution on lung function have been investigated in many cross-sectional and some cohort studies (reviewed by Gotschi et al. 2008). Commonly used lung function measures are forced expiratory volume in 1 sec (FEV_1_), forced vital capacity (FVC), and peak expiratory flow (PEF). A recent review concluded that overall there is evidence for small adverse effects of outdoor air pollution on lung function of children (Gotschi et al. 2008). At present, quantitative summaries of the estimated effects of air pollution on lung function are lacking due to large differences among studies regarding study design, exposure assessment methods, air pollutants, lung function measures, and statistical analysis methods (Gotschi et al. 2008).

A limitation of many studies that have been performed so far is the exposure assessment. Only a few studies have estimated exposure at the individual level; most studies assigned exposures at the community-level without taking into account traffic as a major local source (Gotschi et al. 2008). Moreover, only two studies have investigated exposures at different time points. Early exposure (during the first year of life) and lifetime exposure to nitrogen dioxide (NO_2_) and particulate matter with aerodynamic diameters < 2.5 μm (PM_2.5_) and < 10 μm (PM_10_) were negatively associated with lung function parameters among 9- and 10-year-olds in one study (Oftedal et al. 2008). In the second study, lung function at 8 years of age was associated with traffic PM_10_ exposure during the first year of life, but not with later exposure (Schultz et al. 2012). It is therefore still unclear whether exposure early in life, when the lungs are believed to be more susceptible to environmental exposures, is more relevant to health outcomes than exposure later in life.

In the present study we investigated associations between individual estimates of residential long-term exposure to air pollution and lung function in five European birth cohorts. In the framework of the collaborative European Study of Cohorts for Air Pollution Effects (ESCAPE), a standardized exposure assessment was added to these cohorts. Data were first analyzed on the cohort level following a common protocol, and then cohort-specific effect estimates were combined by random-effects meta-analysis. Individual estimates of early exposure (at the birth address) and current residential exposure from land-use regression (LUR) modeling were available, thus enabling us to estimate effects of exposures at different time points.

## Materials and Methods

*Study population*. This study is a collaborative study of five European birth cohort studies performed in Stockholm county, Sweden [BAMSE: Barn, Allergy, Milieu, Stockholm, Epidemiology (Wickman et al. 2002)]; two parts of Germany, the Munich metropolitan area, and the North-Western part of North-Rhine Westphalia (Ruhr Area), referred to as “South” and “North,” respectively [GINIplus: German Infant Nutrition Intervention study–plus influence of pollution and genetics (Zirngibl et al. 2002) and LISAplus: influence of Life-style related factors on the Immune System and the development of Allergies in childhood–plus the influence of traffic emissions and genetics (Heinrich et al. 2002)]; the greater Manchester area, United Kingdom [MAAS: Manchester Asthma and Allergy Study (Custovic et al. 2002)]; and a series of communities in the north, west, and center of the Netherlands [PIAMA: Prevention and Incidence of Asthma and Mite Allergy (Brunekreef et al. 2002)]. No lung function measurements were performed in LISA South. All studies were designed to study the development of asthma and allergies. Part of the GINI, MAAS, and PIAMA studies were designed as intervention studies. Study participants were born between 1994 (BAMSE) and 1999 (LISA). More information about the study designs and populations is provided in Supplemental Material (see “Study designs and populations,” p. 4, and Figure S1). Ethics approval was obtained from the local authorized institutional review boards, and written informed consent was obtained from the parents or legal guardians of all participants.

The present analysis included participants from these cohorts with successful lung function measurements at 6–8 years of age; complete information on sex, age, height, and weight at the time of lung function measurement; and information on exposure to air pollution at birth and/or the time of lung function measurement (see Supplemental Material, Figure S1).

*Lung function measurements*. In the original cohorts, lung function testing was performed by trained personnel when the children were approximately 6 (GINI and LISA) and 8 years old (BAMSE, PIAMA, MAAS) as described in Supplemental Material (“Lung function measurements,” p. 5). We investigated the following lung function parameters: FEV_1_, FVC, and PEF. Because 6-year-old children can usually perform reliable spirometry but often have short expiratory times, FEV_1_ cannot always be determined. For the younger cohorts (GINI and LISA), we therefore used forced expiratory volume in 0.5 sec (FEV_0.5_), which could be determined for all children, instead of FEV_1_. In addition, FVC is not available for the GINI and LISA cohorts because young children often have difficulties fulfilling the guidelines concerning FVC; and PEF measurements, which also are difficult for young children to perform, failed in almost 20% of the GINI and LISA participants. FEV_1_ (or FEV_0.5_) is the lung function parameter of primary interest because it is available for all cohorts. In all cohorts, body weight and height were measured during the medical examination by trained research staff using calibrated equipment.

*Long-term air pollution exposure assessment*. Annual average air pollution concentrations at each participant’s birth address and current (at time of lung function measurements) home address were estimated by LUR models, as described elsewhere (Beelen et al. 2013; Eeftens et al. 2012a). In brief, air pollution monitoring campaigns were performed between October 2008 and February 2010 in each study area. Three 2-week measurements of NO_2_ and nitrogen oxides (NO_x_) were performed within 1 year at 80 sites in the Netherlands and Belgium and 40 sites in the other areas. Simultaneous measurements of “soot” (determined as the reflectance of PM_2.5_ filters), PM_2.5_, PM_10_, and PM_coarse_ (PM_10_–PM_2.5_) were performed at half of the sites (Cyrys et al. 2012; Eeftens et al. 2012b). Results from the three measurements were averaged to estimate the annual average concentration of each pollutant (Eeftens et al. 2012b). Variables on nearby traffic, population/household density, and land use derived from geographic information systems (GIS) were evaluated as predictors of the spatial variation in annual average concentrations. Regression models were developed to maximize the adjusted explained variance, using a supervised forward stepwise approach. LUR models were then used to estimate annual average air pollution concentrations at the participants’ addresses, for which the same GIS predictor variables were collected. More details are provided in Supplemental Material (“Long-term air pollution exposure assessment,” p. 9). Overall model performance was evaluated by leave-one-out cross-validation: Each site was sequentially left out from the model while the included variables were left unchanged. Leave-one-out cross validation coefficient of determination (*R*^2^) and root mean square errors of the models used for exposure estimation are presented in Supplemental Material, Table S1.

The estimated annual average air pollution concentrations from the LUR models were our primary estimates of exposure. Because air pollution measurements were performed in 2008–2010, but cohort participants were born between 1994 and 1999, we also back-extrapolated predicted concentrations for the birth addresses (largest time difference with the ESCAPE measurements, BAMSE: 12–15 years; GINI South: 10–14 years; GINI/LISA North: 9–14 years; MAAS: 12–15 years; PIAMA: 13–15 years) to account for long-term changes in air pollution levels. Specifically, we used the absolute difference and the ratio between the year before and after birth and the ESCAPE monitoring year, based on data from routine background monitoring network sites in the study areas (for details, see ESCAPE 2013). We used data from 2 years to prevent back-extrapolation from being influenced too much by specific weather circumstances in a specific year. Because routine monitoring data were available only for NO_2_, and PM_10_ in all study areas, back-extrapolation was limited to these pollutants. We did not back-extrapolate exposures for the current addresses (time differences with the ESCAPE measurements were as follows: BAMSE: 4–7 years; GINI South: 4–8 years; GINI/LISA North: 3–8 years; MAAS: 4–7 years; PIAMA: 5–7 years).

In addition to predicted concentrations, traffic intensity on the nearest road (vehicles per day), and total traffic load (vehicle-kilometers driven per day) on all major roads within a 100-m buffer, were used as indicators of exposure and analyzed together with modeled NO_2_ background concentrations.

*Short-term air pollution exposure assessment*. We used routine data from regional and urban background sites of air quality monitoring networks in the study areas to estimate for each participant average exposure to PM, NO_x_, NO_2_, and black smoke on the days preceding the lung function tests. For each participant we used data from the monitoring site that was closest to his or her home. In short-term effect studies, very often the largest effects have been reported for air pollution levels on the day the lung function measurements were performed or on the previous day. However, associations with air pollution for longer lags of up to 5 days have also been reported (Ward and Ayres 2004). We therefore decided to use a 1-week average to avoid missing the potential effects of longer lags. Information on short-term exposures was not available for all pollutants. Therefore, if data were available only for NO_2_ and not for NO_x_, we adjusted long-term NO_x_ models for short-term NO_2_; and if short-term exposures were available for one PM matrix only (e.g., only PM_10_), we adjusted all long-term PM models for that PM matrix. This can be justified by high temporal correlations between the different components.

*Covariates and effect modifiers*. In all cohorts, information on important covariates such as sex, parental socioeconomic status, native ethnicity/nationality, parental allergies, older siblings, any breastfeeding for ≥ 12 weeks, maternal smoking during pregnancy, smoking at the child’s home, mold/dampness in the child’s home, furry pets in the child’s home, use of natural gas for cooking, child-care center attendance during the second year of life, and birth weight was collected by means of questionnaires. Covariates were defined as similarly as possible given the available information. Time-varying covariates were defined for the first year of life and the age at which the lung function measurements were taken, to coincide as much as possible with the air pollution exposure, which was estimated for birth addresses and current addresses.

Asthma and allergic sensitization at the time of lung function measurements (as separate variables), sex, and parental allergy were considered as potential effect modifiers. Asthma at the time of lung function measurements was defined as at least two positive answers to the questions in the 6-year (GINI and LISA) or 8-year questionnaire (BAMSE, PIAMA and MAAS): “Has a doctor ever diagnosed asthma in your child?” “Has your child had wheezing or whistling in the chest in the last 12 months?” “Has your child been prescribed asthma medication during the last 12 months?” Allergic sensitization was defined as specific IgE antibodies of ≥ 0.35 kUA/L for any allergen tested (for details on the allergens and assays used for each cohort, see Supplemental Material, “Definition of allergic sensitization,” p. 9).

*Statistical analysis*. We used a two-stage approach to estimate associations between long-term exposure to air pollution and lung function. First, associations were analyzed on the cohort level. Second, cohort-specific effect estimates were combined by random-effects meta-analysis (DerSimonian and Laird 1986). Because separate LUR models were used for the two subcohorts of the GINI study (South and North), we analyzed the two subcohorts separately to avoid systematic differences in estimated exposures affecting the results. We pooled the GINI North and LISA North cohorts because exactly the same procedures were followed in these cohorts and the same LUR models were used.

We used linear regression analyses with natural log (ln)–transformed lung function parameters as dependent variables to analyze associations between air pollution and continuous lung function parameters (Moshammer et al. 2006). Adjustment of first-stage models for different sets of potential confounders were explored: *a*) Crude models were adjusted for sex, ln(age), ln(weight), and ln(height); because there was no statistically significant interaction between sex and ln(height), no interaction term was included; *b*) adjusted models also included variables that were significantly associated (*p* < 0.05) with lung function in at least one of the cohorts, and that were not on the pathway between air pollution and lung function—ethnicity; parental allergies; parental education; breastfeeding; maternal smoking during pregnancy; smoking, mold/dampness, and furry pets in the child’s home; recent respiratory infections; and study region (BAMSE only, because study region is a design variable in BAMSE that was found to be an important confounder in other analyses); and *c*) extended-adjustment models that also included birth weight, older siblings, use of gas for cooking, child care attendance, and study arm (interventional/observational, where applicable); and models that also included short-term air pollution exposures. In addition, logistic regression analyses were performed to estimate associations between air pollution exposures and clinically low lung function, defined as FEV_1_ < 85% of the cohort-specific predicted value according to sex, age, height, and weight (Moshammer et al. 2006).

As part of a sensitivity analysis, we explored spatial clustering of observations by adding random area-level intercepts (BAMSE: neighborhood and community; GINI/LISA: ZIP-code and community; MAAS: no area-level variable available with sufficient number of children per level; PIAMA: neighborhood, community, region) to the adjusted models. Furthermore, analyses of associations with exposures at the birth address were repeated using back-extrapolated exposure estimates. We explored potential effect modification by asthma and allergic sensitization (both assessed at the time of lung function testing), sex, parental allergy, and moving (defined as any change of address since birth) in stratified analyses on the cohort level, followed by a random-effects meta-analysis. In addition, cohort-specific models with interaction terms were run, and the combined interaction terms from random-effects meta-analyses were tested for statistical significance. Further, we performed two-pollutant models for pollutants that were significantly (*p* < 0.05) associated with FEV_1_ in one-pollutant models. Because NO_2_ and NO_x_ were highly correlated in all cohorts, only NO_2_ was considered.

Effect estimates are presented as the percent-change in each lung function parameter (linear regression) or the odds ratio (OR) for clinically low FEV_1_ (logistic regression), with 95% CIs, for a given increase in exposure (10 μg/m^3^ for NO_2_ and PM_10_, 20 μg/m^3^ for NO_x_, 1 10^–5^/m for PM_2.5_ absorbance, 5 μg/m^3^ for PM_2.5_ and PM_coarse_, 5,000 vehicles/day for traffic intensity on the nearest street, and 4,000 vehicle-km/day for traffic load on major roads within a 100-m buffer). Statistical significance was defined by a two-sided α-level ≤ 5%. Heterogeneity among cohort-specific effect estimates was evaluated with the *I*^2^ statistic (Higgins and Thompson 2002).

## Results

*Characteristics of the study population*. The study population for the present analysis consisted of 5,921 children 6–8 years of age. Characteristics of the study populations and distributions of lung function parameters are presented in Tables 1 and 2, respectively. Population characteristics of the baseline cohorts are presented in Supplemental Material, Table S2. In all cohorts except MAAS, children with highly educated parents and with allergic parents were somewhat overrepresented in the analysis population compared with the baseline population.

**Table 1:** Population characteristics.

Variable	BAMSE (*N* = 2,591)		GINI South (*N* = 653)		GINI/LISA North (*N* = 958)		MAAS (*N* = 661)		PIAMA (*N* = 1,058)	
	*n*/*N*	Percent	*n*/*N*	Percent	*n*/*N*	Percent	*n*/*N*	Percent	*n*/*N*	Percent
Female sex	1,268/2,591	48.9	337/653	51.6	479/958	50.0	310/661	46.9	533/1,058	50.4
Respiratory infections^*a*^	236/2,592	9.1	227/650	34.9	373/938	39.8	0/661	0.0	253/1,054	24.2
Allergic mother	432/2,563	16.9	373/653	57.1	352/955	36.9	386/645	59.8	699/1,058	66.1
Allergic father	460/2,563	18.0	326/647	50.4	287/950	30.2	402/641	62.7	351/1,055	33.3
Current asthma^*b*^	263/2,588	10.2	25/653	3.8	41/950	4.3	118/659	17.9	105/990	10.6
Allergic sensitization^*b*^	851/2,447	34.8	228/596	38.3	246/842	29.2	180/406	44.3	395/869	45.5
Native ethnicity/nationality^*c*^	2,023/2,576	78.5	653/653	100.0	958/958	100.0	623/655	95.1	990/1,044	95.7
High maternal SES^*d*^	1,083/2,579	42.0	381/652	58.4	338/955	35.4	NA		407/1,055	38.6
High paternal SES^*d*^	1,000/2,532	39.9	440/647	68.0	374/949	39.4	106/608	17.4	447/1,043	42.9
Older siblings	1,228/2,591	47.4	258/651	39.6	511/955	53.5	324/643	50.4	509/1,058	48.1
Breastfeeding (≥ 12 weeks)	2,397/2,516	95.3	445/640	69.5	526/924	56.9	307/630	48.7	556/1,058	52.6
Mother smoked during pregnancy	311/2,590	12.0	85/646	13.2	131/944	13.9	73/659	11.1	161/1,044	15.4
Smoking at child’s home
Early life	524/2,578	20.3	102/642	15.9	255/944	27.0	277/658	42.1	266/1,058	25.1
Current^*b*^	468/2,549	18.4	133/653	20.4	344/953	36.1	241/649	37.1	155/990	15.7
Use of natural gas for cooking
Early life	285/2,591	11.0	44/643	6.8	47/938	5.0	520/660	78.8	875/1,053	83.1
Current^*b*^	185/2,584	7.2	43/653	6.8	37/948	3.9	529/661	80.0	801/1,047	76.5
Mold/dampness in child’s home
Early life	653/2,582	25.3	204/643	31.7	199/937	21.2	116/661	17.5	297/1,042	28.5
Current^*b*^	254/2,579	9.9	158/652	24.2	176/936	18.8	102/661	15.4	284/985	28.8
Furry pets in home
Early life	382/2,591	14.7	95/634	15.0	152/922	16.5	243/661	36.8	454/1,056	43.0
Current^*b*^	647/2,583	25.1	157/652	24.2	253/951	26.6	289/661	43.7	484/970	49.9
Child-care center attendance^*e*^	2,148/2,539	84.6	51/619	8.2	13/880	1.5	431/621	69.4	289/1,032	28.0
Study arm
Observational cohort	NA		247/653	37.8	575/958	60.0	579/661	87.6	615/1,048	58.7
Intervention group	NA		406/653	62.2	383/958	40.0	82/661	12.4	433/1,048	41.4
Moved^*f*^	1,644/2,538	64.8	340/631	53.9	323/952	33.9	365/661	55.2	551/1,058	52.4
Birth weight (g) (mean ± SD)^*g*^	3,530 ± 559	2,498	3,421 ± 446	644	3,532 ± 495	932	3,484 ± 501	634	3,508 ± 548	1,056
NA, not applicable/not available. ^***a***^BAMSE and MAAS: Respiratory infection at time of lung function measurement; GINI and LISA: Lower or upper respiratory infection during past 4 weeks; PIAMA: Cold or respiratory infection during past 3 weeks. ^***b***^At the age of lung function testing. ^***c***^BAMSE: Scandinavian; GINI and LISA: German; MAAS: Caucasian; PIAMA: Dutch. ^***d***^SES, socioeconomic status; defined by education for BAMSE, GINI and LISA, and PIAMA and by income (> £ 30,000) in MAAS. ^***e***^During second year of life. ^***f***^Any change of address between birth and lung function measurement. ^***g***^*N* = 2,498; the values in the adjacent “Percent” columns are *n*s.										

**Table 2:** Lung function measurements and the prevalence of low lung function according to cohort.

Variable	BAMSE		GINI South		GINI/LISA North		MAAS		PIAMA	
	Mean ± SD or *n* (%)	*N*	Mean ± SD or *n* (%)	*N*	Mean ± SD or *n* (%)	*N*	Mean ± SD or *n* (%)	*N*	Mean ± SD or *n* (%)	*N*
FEV_1_ (L)^*a*^	1.78 ± 0.27	2,027	1.09 ± 0.16	653	1.10 ± 0.16	958	1.59 ± 0.25	661	1.80 ± 0.25	1,058
FVC (L)	2.07 ± 0.33	2,057	—	—	—	—	1.83 ± 0.28	661	2.01 ± 0.30	1,058
PEF (L/sec)	4.85 ± 0.69	2,555	3.10 ± 0.53	540	3.04 ± 0.52	773	—	—	3.79 ± 0.63	1,058
Height (cm)	132.2 ± 6.1	2,591	119.4 ± 4.6	653	121.1 ± 5.1	958	128.3 ± 5.6	661	132.9 ± 5.6	1,058
Weight (kg)	30.2 ± 5.5	2,591	21.9 ± 2.9	653	23.5 ± 3.6	958	28.4 ± 5.7	661	28.9 ± 4.8	1,058
Age (years)	8.3 ± 0.5	2,591	6.1 ± 0.1	653	6.3 ± 0.2	958	8.0 ± 0.2	661	8.1 ± 0.3	1,058
Low lung function^*b*^	137 (6.8)	2,027	68 (10.4)	653	93 (9.7)	958	51 (7.7)	661	71 (6.7)	1,058
^***a***^FEV_1_ for BAMSE, MAAS, and PIAMA; FEV_0.5_ for GINI and LISA. ^***b***^FEV_1_ (BAMSE, MAAS, and PIAMA) or FEV_0.5_ (GINI and LISA) < 85% predicted based on age, sex, height, and weight.										

*Air pollution exposure*. Distributions of estimated annual average air pollution levels at the birth address and current address, and of short-term air pollution exposures, are presented in Table 3. Mean concentrations of all pollutants except PM_coarse_ were lowest for the Swedish BAMSE cohort. Ranges were larger for NO_x_, NO_2_, and PM_2.5_ absorbance than for particle mass concentrations. Correlations between annual average air pollution levels at the birth address and current address are presented for each cohort in Supplemental Material, Tables S3–S7. NO_2_ and NO_x_ were highly correlated (≥ 0.88) for current and birth addresses in all cohorts except MAAS; NO_2_ and PM_2.5_ absorbance were highly correlated (≥ 0.91) in BAMSE and PIAMA. Correlations between estimated annual average air pollution levels at birth and current addresses for the same pollutant were moderate to high (*r* = 0.26–0.88) depending on the cohort and pollutant. Correlations between pollutants and traffic indicators were mostly moderate or low. There were essentially no correlations between estimated annual average and short-term exposures, except for a few positive correlations for the PIAMA study (e.g., *r* = 0.48 and 0.53 for short-term NO_2_ and NO_2_ at the birth address and current address, respectively) (see Supplemental Material, Table S8).

**Table 3:** Distribution of estimated annual average air pollution levels, traffic indicators, and short-term air pollution exposure variables.

Pollutant	BAMSE		GINI South		GINI/LISA North		MAAS		PIAMA	
	Mean ± SD	Min–Max	Mean ± SD	Min–Max	Mean ± SD	Min–Max	Mean ± SD	Min–Max	Mean ± SD	Min–Max
Birth address
NO_2_ (μg/m^3^)	14.0 ± 5.4	6.0–33.0	21.7 ± 5.9	11.5–61.1	23.7 ± 3.6	19.7–62.8	22.9 ± 2.1	16.0–30.4	23.1 ± 6.7	9.4–59.6
NO_x_ (μg/m^3^)	25.5 ± 12.0	11.5–86.3	36.3 ± 10.2	19.7–121.4	34.5 ± 9.7	23.9–147.7	38.9 ± 5.1	26.1–77.8	34.5 ± 12.4	16.5–98.9
PM_2.5_ abs (10^–5/^m)	0.7 ± 0.2	0.4–1.3	1.7 ± 0.2	1.3–3.6	1.2 ± 0.2	1.0–3.1	1.1 ± 0.2	0.7–1.9	1.2 ± 0.3	0.8–3.0
PM_2.5_ (μg/m^3^)	7.8 ± 1.2	4.2–10.9	13.4 ± 1.0	11.1–17.6	17.4 ± 0.7	15.8–21.5	9.4 ± 0.2	9.4–11.0	16.4 ± 0.7	15.3–21.1
PM_10_ (μg/m^3^)	15.7 ± 3.7	6.0–30.9	20.4 ± 2.4	14.8–34.4	25.4 ± 1.2	23.9–33.4	17.1 ± 0.9	12.6–22.7	25.0 ± 1.2	23.7–33.2
PM_coarse_ (μg/m^3^)	7.9 ± 2.9	0.7–20.2	6.7 ± 1.5	4.1–16.0	8.5 ± 0.7	1.9–13.8	7.0 ± 0.8	5.0–11.5	8.4 ± 0.8	7.6–13.0
NO_2_ background (μg/m^3^)	13.0 ± 3.4	3.6–21.3	20.3 ± 4.1	14.0–31.3	23.7 ± 0.9	22.9–36.3	21.4 ± 1.1	18.0–23.3	21.5 ± 4.9	13.1–35.6
Traffic intensity (veh/day)^*a*^	2,351 ± 4,430	122–52,020	2,518 ± 6,695	500–82,226	1,189 ± 2,499	454–20,726	827 ± 2,163	500–29,590	972 ± 3,241	0–46,121
Traffic load (veh-km/day)^*b*^	971 ± 1,629	0–21,400	1,031 ± 2,543	0–25,364	263 ± 793	0–11,178	763 ± 3,761	0–63,464	592 ± 1,704	0–20,605
Current address
NO_2 _(μg/m^3^)	11.9 ± 5.0	6.0–30.5	20.2 ± 5.1	11.5–55.7	23.4 ± 2.8	19.7–59.8	22.6 ± 2.0	16.0–28.6	22.2 ± 6.3	9.4–52.1
NO_x_ (μg/m^3^)	21.1 ± 10.9	11.5–74.1	34.1 ± 8.5	19.7–110.0	33.6 ± 6.8	23.9–100.3	38.4 ± 5.0	26.4–77.8	32.8 ± 11.2	16.5–100.1
PM_2.5_ abs (10^–5/^m)	0.6 ± 0.2	0.4–1.2	1.7 ± 0.2	1.3–3.4	1.2 ± 0.2	1.0–4.5	1.1 ± 0.2	0.7–1.9	1.2 ± 0.2	0.8–2.1
PM_2.5_ (μg/m^3^)	7.4 ± 1.3	4.2–11.0	13.4 ± 0.9	10.9–18.8	17.3 ± 0.6	15.8–21.4	9.4 ± 0.1	9.4–10.8	16.3 ± 0.7	14.9–19.3
PM_10_ (μg/m^3^)	15.3 ± 3.5	6.0–30.9	20.1 ± 2.3	14.8–30.2	25.3 ± 1.0	23.9–31.4	17.0 ± 0.7	12.6–22.3	24.8 ± 1.1	23.7–29.8
PM_coarse_ (μg/m^3^)	7.6 ± 2.7	0.7–20.2	6.4 ± 1.3	4.1–13.5	8.4 ± 0.6	1.9–13.8	7.0 ± 0.7	5.2–11.3	8.3 ± 0.7	7.6–11.2
NO_2_ background (μg/m^3^)	11.5 ± 3.6	3.6–22.8	19.1 ± 3.8	14.0–31.9	23.7 ± 0.9	22.9–36.3	21.3 ± 1.1	18.1–23.3	21.1 ± 4.7	13.1–35.6
Traffic intensity (veh/day)^*a*^	1,895 ± 4,072	122–50,920	2,022 ± 7,499	500–134,000	1,061 ± 2,128	500–16,806	755 ± 2,099	500–29,590	777 ± 2,731	0–46,121
Traffic load (veh-km/day)^*b*^	689 ± 1,523	0–25,000	752 ± 2,683	0–54,297	256 ± 910	0–16,905	689 ± 3,825	0–63,464	407 ± 1,191	0–14,670
Short-term exposure
NO_2 _(μg/m^3^)	17.5 ± 4.2	9.3–36.9	25.7 ± 8.6	11.0–62.9	24.3 ± 8.7	7.7–61.5	30.7 ± 9.3	11.7–65.0	22.6 ± 10.9	2.7–55.7
NO_x_ (μg/m^3^)	23.0 ± 7.9	11.3–78.2	—	—	—	—	—	—	31.7 ± 20.4	3.7–151.0
Black smoke (μg/m^3^)	—	—	—	—	—	—	—	—	6.6 ± 4.5	0.0–23.1
PM_2.5_ (μg/m^3^)	11.0 ± 4.2	5.7–31.4	—	—	—	—	—	—	—	—
PM_10_ (μg/m^3^)	19.2 ± 7.8	8.9–44.1	33.4 ± 13.4	13.5–86.8	21.5 ± 9.6	6.0–67.6	23.4 ± 5.5	10.3–41.4	28.6 ± 9.7	12.3–69.0
Abbreviations: Max, maximum; Min, minimum; PM_2.5_ abs, PM_2.5_ absorbance; veh, vehicle. ^***a***^On nearest street. ^***b***^On major roads within 100-m buffer.										

**Figure 1:**
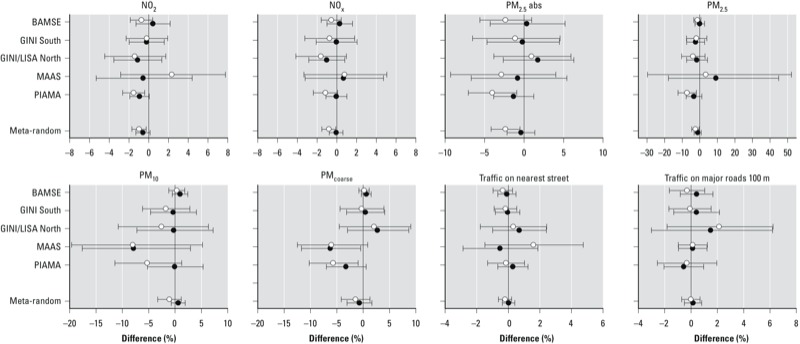
Adjusted center-specific and combined (meta-random) associations of annual average levels of air pollution and traffic indicators with FEV_1_. Error bars are 95% CIs. Associations with exposures at birth address are represented by black dots, and associations with exposures at current address by white dots. Estimates are adjusted for age, sex, height, weight, recent respiratory infections, ethnicity/nationality, parental education, allergic mother, allergic father, breastfeeding, mother smoking during pregnancy, smoking at home, mold/dampness at home, furry pets at home. Associations are presented for the following increments in exposure: 10 μg/m^3^ for NO_2_, 20 μg/m^3^ for NO_x_, 1 unit for PM_2.5_ absorbance (PM_2.5_abs), 5 μg/m^3^ for PM_2.5_, 10 μg/m^3^ for PM_10_, 5 μg/m^3^ for PM_coarse_, 5,000 vehicles/day for traffic intensity on the nearest street; and 4,000 vehicle-km/day for traffic load on major roads within a 100-m buffer; associations with traffic intensity and traffic load were additionally adjusted for background NO_2_ concentrations.

*Associations between air pollution and lung function*. Associations between annual average air pollution levels and lung function from meta-analyses were very similar in the crude and the adjusted models (see Table 4 for FEV_1_; see also Supplemental Material, Tables S9 and S10, for FVC and PEF, respectively). Associations showed little or no heterogeneity among the cohorts for FEV_1_ and PEF, except for associations with PM_coarse_. However, associations with FVC were more heterogeneous. Most associations were negative, suggesting decreases in lung function of a few percent with increasing exposure (see Figure 1 for FEV_1_; see also Supplemental Material, Figures S2 and S3, for FVC and PEF, respectively). Overall, there were statistically significant negative associations between FEV_1_ and NO_2_, NO_x_, PM_2.5_ absorbance, and PM_2.5_ at the current address. Similarly, we estimated statistically significant negative associations for FVC with NO_2_, NO_x_, and PM_2.5_ absorbance at the current address, and for PEF with NO_2_ and PM_2.5_ at the current address. Results remained unchanged in models with extended adjustment (data not shown). Associations of all three lung function parameters and short-term exposure to NO_2_ and PM_10_ were negative, but were not statistically significant (see Supplemental Material, Table S11).

**Table 4:** Crude and adjusted associationsa of annual average levels of air pollution and traffic indicators with FEV_1_: results from random-effects meta-analyses.

Exposure	Crude^*b*^^,^^*c*^		Adjusted^*d*^^,^^*e*^	
	Percent difference (95% CI)	*I*^2^ (*p*_het_)	Percent difference (95% CI)	*I*^2^ (*p*_het_)
Birth address
NO_2_	–0.47 (–1.03, 0.11)	0.0 (0.5646)	–0.59 (–1.31, 0.14)	0.0 (0.7049)
NO_x_	–0.20 (–0.75, 0.35)	0.0 (0.8327)	–0.07 (–0.76, 0.62)	0.0 (0.8272)
PM_2.5_ absorbance	–0.23 (–1.70, 1.26)	0.0 (0.6974)	–0.41 (–2.15, 1.36)	0.0 (0.8211)
PM_2.5_	–0.50 (–2.08, 1.11)	0.0 (0.4887)	–1.22 (–3.20, 0.80)	0.0 (0.6762)
PM_10_	0.28 (–0.86, 1.44)	0.0 (0.9423)	0.59 (–0.72, 1.91)	0.0 (0.5677)
PM_coarse_	–0.72 (–2.92, 1.54)	55.9 (0.0595)	–0.73 (–3.06, 1.66)	56.5 (0.0562)
Traffic intensity nearest street	–0.08 (–0.47, 0.30)	0.0 (0.4523)	0.02 (–0.38, 0.42)	0.0 (0.8631)
Traffic load major roads 100-m buffer	0.21 (–0.41, 0.84)	0.0 (0.9041)	0.15 (–0.50, 0.81)	0.0 (0.8381)
Current address
NO_2_	–1.05 (–1.67,–0.42)	0.0 (0.6444)	–0.98 (–1.70,–0.26)	0.0 (0.5148)
NO_x_	–0.86 (–1.48,–0.24)	0.0 (0.6811)	–0.82 (–1.52,–0.11)	0.0 (0.8331)
PM_2.5_ absorbance	–1.90 (–3.51,–0.26)	0.0 (0.5007)	–2.37 (–4.18,–0.52)	0.0 (0.5319)
PM_2.5_	–1.77 (–3.34,–0.18)	0.0 (0.4589)	–2.49 (–4.57,–0.36)	8.5 (0.3578)
PM_10_	–0.67 (–2.32, 1.02)	8.2 (0.3599)	–1.09 (–3.32, 1.18)	19.2 (0.2923)
PM_coarse_	–1.31 (–3.97, 1.43)	59.6 (0.0422)	–1.47 (–4.14, 1.29)	54.9 (0.0645)
Traffic intensity nearest street	–0.22 (–0.62, 0.17)	0.0 (0.7385)	–0.21 (–0.63, 0.22)	0.0 (0.7795)
Traffic load major roads 100-m buffer	0.06 (–0.61, 0.73)	0.0 (0.5517)	–0.01 (–0.71, 0.69)	0.0 (0.8379)
^***a***^Associations are expressed as percent change with 95% CIs, *I*^2^, and *p*-value of test for heterogeneity (*p*_het_) of effect estimates between cohorts and presented for the following increments in exposure: 10 μg/m^3^ for NO_2_, 20 μg/m^3^ for NO_x_, 1 unit for PM_2.5_ absorbance, 5 μg/m^3^ for PM_2.5_, 10 μg/m^3^ for PM_10_, 5 μg/m^3^ for PM_coarse_, 5,000 vehicles/day for traffic intensity on the nearest street; and 4,000 vehicle-km/day for traffic load on major roads within a 100-m buffer. ^***b***^Adjusted for age, sex, height, and weight all participants; associations with traffic intensity and traffic load were additionally adjusted for background NO_2_ concentrations. ^***c***^*N* = 5,317 for birth address and 5,169 for current address. ^***d***^Additionally adjusted for recent respiratory infections, ethnicity/nationality, parental education, allergic mother, allergic father, breastfeeding, mother smoking during pregnancy, smoking at home, mold/dampness at home, furry pets at home, and study region (BAMSE only). ^***e***^*N* = 4,887 for birth address and 4,656 for current address.				

Logistic regression analyses showed significant positive associations between clinically low lung function (FEV_1_ < 85% predicted) and annual average levels of NO_2,_ NO_x_, PM_2.5_ absorbance, and PM_10_ at the current address (Table 5).

**Table 5:** Adjusted^*a*^ associations^*b*^ of annual average levels of air pollution and traffic indicators at the current address with low lung function (FEV_1_ < 85% predicted): results from random-effects meta-analyses.

Exposure	OR (95% CI)	*I*^2^ (*p*_het_)
NO_2_	1.35 (1.06,1.73)	0.0 (0.6391)
NO_x_	1.33 (1.05,1.69)	0.0 (0.5934)
PM_2.5_ absorbance	1.85 (1.00,3.43)	0.0 (0.6426)
PM_2.5_	1.41 (0.74,2.71)	0.0 (0.4194)
PM_10_	1.69 (1.04,2.74)	0.0 (0.9111)
PM _coarse_	1.81 (0.94,3.47)	0.0 (0.5403)
Traffic intensity nearest street	1.05 (0.92,1.20)	0.0 (0.7797)
Traffic load major roads 100-m buffer	1.00 (0.97,1.03)	0.0 (0.9571)
^***a***^Adjusted for recent respiratory infections, ethnicity/nationality, parental education, allergic mother, allergic father, breastfeeding, mother smoking during pregnancy, smoking at home, mold/dampness at home, furry pets at home, and study region (BAMSE only); associations with traffic intensity and traffic load were additionally adjusted for background NO_2_ concentrations. ^***b***^Associations are expressed as ORs with 95% CIs, *I*^2^, and *p*-value of test for heterogeneity (*p*_het_) of effect estimates between cohorts and presented for the following increments in exposure: 10 μg/m^3^ for NO_2_, 20 μg/m^3^ for NO_x_, 1 unit for PM_2.5_ absorbance, 5 μg/m^3^ for PM_2.5_, 10 μg/m^3^ for PM_10_, 5 μg/m^3^ for PM_coarse_, 5,000 vehicles/day for traffic intensity on the nearest street; and 4,000 vehicle-km/day for traffic load on major roads within a 100-m buffer.		

*Sensitivity analyses*. We found little indication of spatial clustering of observations. For all but two exposure–outcome combinations (PIAMA: PEF and PM_10_ at current and birth address) random area-level intercepts were statistically nonsignificant (data not shown).

Stratified analyses did not reveal systematically different associations for asthmatic and nonasthmatic children, for sensitized and nonsensitized children, for girls and boys, and for children of allergic and nonallergic parents (see Supplemental Material, Tables S12–S15, respectively). Associations with annual average PM_10_ and PM_coarse_ tended to be stronger in asthmatic than in nonasthmatic children, and associations with annual average PM_2.5_ absorbance and PM_2.5_ tended to be somewhat stronger in boys compared with girls, but confidence intervals largely overlapped, and none of the interaction terms was statistically significant. For all pollutants, associations with exposures at the current address tended to be stronger for children who moved residence after birth than for children who did not move (see Supplemental Material, Table S16).

Associations with annual average air pollution levels at the birth address were not substantially different for exposures that were estimated using back-extrapolation to the children’s birth years (see Supplemental Material, Table S17). Results for two-pollutant models that included NO_2_ and PM_2.5_ were mixed: Although mutual adjustment moved all estimates closer to the null, for FEV_1_ and PEF associations with NO_2_ decreased (relatively) more than associations with PM_2.5_, whereas for FVC the decrease was more pronounced for the association with PM_2.5_ than with NO_2_ (see Supplemental Material, Table S18). Two-pollutant models with NO_2_ and PM_2.5_ absorbance resulted in multicollinearity problems in BAMSE and PIAMA (variance inflation factor > 5) and are therefore not presented.

## Discussion

Estimated long-term exposures to NO_2_, NO_x_, PM_2.5_ absorbance, and PM_2.5_ at the current address were associated with decreases in lung function in five European birth cohort studies. Estimated effects of long-term exposures did not appear to be confounded by short-term exposures to the same pollutants.

The present analysis extends previous work within two of the participating cohorts, in which associations of air pollution with interrupter resistance, a technique that measures the resistance of the respiratory system (Eenhuizen et al. 2013), and PEF at 4 years of age (Nordling et al. 2008), and with FEV_1_ at 8 years (Schultz et al. 2012) were found. Comparisons of our findings with those of other studies are limited by the great diversity in study designs, exposure assessments, lung function measures, and statistical methods used. However, overall, our finding of a small decrease in lung function with increasing exposure to air pollution is consistent with the findings of other studies in schoolchildren that have compared individuals within communities. For example, when estimates are rescaled to the exposure contrasts used in the present analysis, statistically significant decreases in PEF ranging from 0.8% per 5-μg/m^3^ increase in PM_2.5_ to 3.2% per 10-μg/m^3^ increase in NO_2_ were estimated for a Norwegian study population (Oftedal et al. 2008), and a decrease of 4.8% in FEV_1_ per 10-μg/m^3^ increase in traffic-PM_10_ was estimated in the BAMSE cohort (Schultz et al. 2012).

Automobile traffic was associated with decreases in FEV_1_ and PEF corresponding to < 0.1% per 5,000 cars/day in a German study (Wjst et al. 1993), and truck traffic, but not all traffic, was associated with decreases in lung function ranging from 0.4% for FVC to 1.7% for PEF, per 5,000 trucks/day, in a Dutch study (Brunekreef et al. 1997). No association was found between the two traffic indicator variables and lung function in the present study. One potential explanation may be that we could not differentiate between truck and automobile traffic in the present study. Another potential explanation may be that although traffic is an important source of air pollution in the study areas, it is not the only source. Industry (GINI/LISA North and MAAS) and ports (GINI/LISA North and PIAMA), for example, were additional determinants of air pollution levels in some of the areas (Beelen et al. 2013; Eeftens et al. 2012a).

Our analyses, which are based on a standardized exposure assessment and common analysis protocol, revealed little heterogeneity of the associations between air pollution and FEV_1_ and PEF between cohorts. With five studies, however, statistical power to detect heterogeneity in results among the birth cohorts is limited. In the present study, lung function was associated with NO_2_, NO_x_, PM_2.5_, and PM_2.5_ absorbance, but not with PM_10_ or PM_coarse_. Effects were observed in study populations with exposures that were well below the current European air quality limit values (European Commission 2013). Although the estimated decreases in lung function due to air pollution are small on the population level, they were associated with significant increases in prevalence of low lung function (based on FEV_1_ < 85% of predicted values). Prospective cohort studies following children and adolescents into early adulthood are needed to investigate whether early deficits in lung function will be compensated for by a longer growth phase, or whether these subjects will enter the lung-function decline phase of later adulthood with a reduced lung function (Gotschi et al. 2008).

Oxidative stress and inflammation have been hypothesized as the main mechanisms through which ambient air pollution can affect human health. With regard to lung function, toxicological evidence on mechanisms is sparse [HEI (Health Effects Institute) Panel on the Health Effects of Traffic-Related Air Pollution 2010]. Some evidence comes from a study in Mexican schoolchildren that showed that exposure to PM_2.5_ is associated with both acute airway inflammation and decreased lung function (Barraza-Villarreal et al. 2008).

So far, only two studies investigated the role of exposure at different time points. Oftedal et al. (2008) reported that lung function in 9- and 10-year-old children was associated with exposure during the first year of life and lifetime exposure, whereas in the BAMSE cohort, lung function at 8 years of age was associated with exposure during the first year of life, but not with later exposure (Schultz et al. 2012). Findings of the present study indicate stronger associations with current exposure than with early-life exposures (estimated for the address at birth), including associations estimated for children in the BAMSE cohort. The possibility that current exposures may be more relevant than early-life exposures to lung function is supported by the findings from studies suggesting that air pollution effects on lung function in children may be reversible (Avol et al. 2001; Rojas-Martinez et al. 2007). However, measurement error could be at least partly responsible for the stronger associations with exposures at the current addresses because measurement error associated with LUR estimation of historical exposures likely increases with increasing time difference. We used data from measurements performed in 2008–2010 to build our exposure models, and applied them to the children’s historical addresses—implicitly assuming that the spatial variability would not have changed since the baseline time period for each cohort (1994–1999). Likewise, an underlying assumption of our back-extrapolation procedure is that spatial patterns remain constant over time. Evidence supporting this assumption is provided by three studies that reported that spatial contrasts in measured and modeled annual average NO_2_ concentrations were stable over 7–12 years (Cesaroni et al. 2012; Eeftens et al. 2011; Wang et al. 2013). One of these studies, from Vancouver, British Columbia, Canada, reported that LUR models were better at forecasting than at backcasting over a 7-year period, with forecasting *R*^2^ of 0.52–0.61 for NO_2_ and backcasting *R*^2^ of 0.44–0.49 (Wang et al. 2013); this might explain the lack of association with exposures at the current address. A study from Rome, Italy, reported that a LUR model developed with NO_2_ measurements conducted in 2007 was better at explaining the spatial variation of measurements conducted in 1995–1996 (*R*^2^ = 0.69) than the 1995–1996 model was at explaining the variation in 2007 measurements (*R*^2^ = 0.53) (Cesaroni et al. 2012). A Dutch study (Eeftens et al. 2011) reported very high agreement for backcasting from 2007 to 1999 (*R*^2^ = 0.77) as well as for forecasting from 1999 to 2007 (*R*^2^ = 0.81). Because time differences with ESCAPE monitoring campaigns for birth and current addresses were in the same range for the different cohorts, we do not expect that time differences would have influenced the cohort-specific findings differentially.

Separate analyses in movers and nonmovers suggested stronger effects in movers. Differences between strata, however, were not statistically significant. One possible explanation could be that families of sensitive children tend to move to places with less traffic exposure. However, because very few children fell into this category, this is unlikely to explain our finding.

Whether the susceptibility to the effects of air pollution differs between boys and girls remains unclear. In our study we did not observe significant differences or consistent patterns in associations between boys and girls. Several other studies reported stronger associations for girls (Frye et al. 2003; Oftedal et al. 2008; Peters et al. 1999), whereas others reported stronger associations for boys (Brunekreef et al. 1997; Schultz et al. 2012), or no differences (Raizenne et al. 1996).

An important question concerns the issue of pollutant-specific effects: Which (set of) pollutant(s) is responsible for the observed effects? Two-pollutant models with NO_2_ and PM_2.5_ were inconclusive, and it was not possible to disentangle the effects of NO_2_ and PM_2.5_ absorbance due to high correlations in some of the cohorts.

Use of common exposure assessment and statistical analysis protocols across multiple cohorts is an important strength of our study. Another advantage of our study, which uses data from prospective birth cohort studies, over cross-sectional studies is the availability of the participants’ residential histories; this allows us to investigate the effect of exposure at different time points and potential effect modification by moving. In all cohorts included in the present analysis, except MAAS, children with highly educated parents and with allergic parents were overrepresented in the analysis population compared with the baseline populations, either by design or because of differential loss to follow up. Therefore, the generalizability of the present findings to the original cohorts, and to the general population, may be limited. Another limitation may be that exposure was defined as exposure at the participants’ residential address, and that time–activity patterns and exposures at nonresidential addresses, like child-care centers or schools, were not accounted for. However, in the BAMSE study the correlation between estimated exposures based only on residential addresses and those based on home addresses and other locations were found to be high; consequently, associations with health outcomes were not substantially different (Gruzieva et al. 2012).

## Conclusion

Our findings suggest that exposure to air pollution may result in reductions in lung function in schoolchildren. Although estimated changes in lung function parameters were relatively small, our results suggest the possibility that exposure may increase the prevalence of clinically relevant declines in lung function in the population as a whole.

## Supplemental Material

(1.1 MB) PDF
