# Supplementary material for: Air Pollution Exposure and Lung Function in Children: The ESCAPE Project
Source: Environ Health Perspect. 2013 Sep 27;121(11-12):1357–64. doi: 10.1289/ehp.1306770 (PMC3855518; doi:10.1289/ehp.1306770)
Supplement: (1.1 MB) PDF [file ehp.1306770.s001.508.pdf]

Environmental Health Perspectives doi:10.1289/ehp.1306770

Supplemental Material

Air Pollution Exposure and Lung Function in Children: The ESCAPE Project

Ulrike Gehring, Olena Gruzieva, Raymond M. Agius, Rob Beelen, Adnan Custovic, Josef Cyrys, Marloes Eeftens, Claudia Flexeder, Elaine Fuertes, Joachim Heinrich, Barbara Hoffmann, Johan C. de Jongste, Marjan Kerkhof, Claudia Klümper, Michal Korek, Anna Mölter, Erica Schultz, Angela Simpson, Dorothea Sugiri, Magnus Svartengren, Andrea von Berg, Alet H. Wijga, Göran Pershagen, and Bert Brunekreef

Table of contents

Section, Table, or Figure	Page
Study designs and study populations	4
Lung function measurements	5
Long-term air pollution exposure assessment	9
Definition of allergic sensitization	9
References	11
**Supplemental Material, Table S1.** Performance of the Land-use regression models used for exposure estimation: leave-one-out cross-validation R^2 (R^2_{CV}) and root mean square error (RMSE) [µg/m^3].	12
**Supplemental Material, Table S2.** Population characteristics of the baseline birth cohorts.	13
**Supplemental Material, Table S3.** Pearson correlations between long-term air pollution exposure estimates at the current and birth address for the BAMSE cohort.	14
**Supplemental Material, Table S4.** Pearson correlations between long-term air pollution exposure estimates at the current and birth address for the GINI South cohort.	15
**Supplemental Material, Table S5.** Pearson correlations between long-term air pollution exposure estimates at the current and birth address for the GINI/LISA North cohort.	16
**Supplemental Material, Table S6.** Pearson correlations between long-term air pollution exposure estimates at the current and birth address for the MAAS cohort.	17
**Supplemental Material, Table S7.** Pearson correlations between long-term air pollution exposure estimates at the current and birth address for the PIAMA cohort.	18
**Supplemental Material, Table S8.** Pearson correlations between short-term exposures and estimated annual average air pollution levels at the birth and current address.	19
**Supplemental Material, Table S9**. Crude and adjusted associations of annual average air pollution levels and traffic indicators with FVC: results from random-effects meta-analyses.	20
**Supplemental Material, Table S10**. Crude and adjusted associations of annual average air pollution levels and traffic indicators with PEF: results from random-effects meta-analyses.	22
**Supplemental Material, Table S11.** Adjusted associations of average air pollution levels on the seven days preceding the lung function measurements with lung function: results from random-effects meta-analyses.	24

Section, Table, or Figure	Page
**Supplemental Material, Table S12**. Adjusted associations of annual average air pollution levels and traffic-indicators at the current address with FEV_1 for asthmatic and non-asthmatic children separately: results from random-effects meta-analyses.	25
**Supplemental Material, Table S13**. Adjusted associations of annual average air pollution levels and traffic-indicators at the current address with FEV_1 for sensitized and non-sensitized children separately: results from random-effects meta-analyses.	26
**Supplemental Material, Table S14**. Adjusted associations of annual average air pollution levels and traffic indicators at the current address with FEV_1 for boys and girls separately: results from random-effects meta-analyses.	27
**Supplemental Material, Table S15**. Adjusted associations of annual average air pollution levels and traffic indicators at the current address with FEV_1 for children with and without allergic parents separately: results from random-effects meta-analyses.	28
**Supplemental Material, Table S16**. Adjusted associations of annual average air pollution levels and traffic indicators at the current address with FEV_1 stratified by moving between birth and lung function measurements: results from random-effects meta-analyses.	29
**Supplemental Material, Table S17**. Adjusted associations of back-extrapolated levels of NO_x, NO_2, and PM_{10} at the birth address with lung function: results from random-effects meta-analyses.	30
**Supplemental Material, Table S18.** Adjusted associations of annual average levels of air pollution with lung function from two-pollutant models with NO_2 and $PM_{2.5}$: results from random-effects meta-analyses.	31
**Supplemental Material, Figure S1.** Study population.	32
**Supplemental Material, Figure S2.** Forest plots of adjusted center-specific and combined associations of annual average air pollution levels and traffic indicators with FVC.	33
**Supplemental Material, Figure S3.** Forest plots of adjusted center-specific and combined associations of annual average air pollution levels and traffic indicators with PEF.	34

Materials and Methods

Study designs and study populations

For the BAMSE birth cohort study, between February 1994 and November 1996, 4089 newborn infants were recruited from Child Health Centers. The study population comprised 75% of all eligible children born in 4 predefined areas of central and north-western parts of Stockholm, representing urban and suburban environments.

For the GINIplus birth cohort study, a total of 5991 newborns were recruited across the two German cities of Munich (2949 children) and Wesel (3042 children) from 1995 to 1998. Children with a family history of allergy (N=2252) had the opportunity to participate in a prospective, double-blinded nutritional intervention aimed at assessing the effect of different baby formulas on allergy development. Children without a family history of allergy (or those who declined to participate in the intervention) were assigned to the non-intervention group (N=3739).

For the LISAplus birth cohort study, neonates were recruited from four German cities of Munich, Wesel, Leipzig and Bad Honnef, Munich (West Germany) and Leipzig (East Germany). Recruitment took place in obstetric clinics shortly after birth. From December 1997 to January 1999, the target population of the study was defined as newborns from parents who were born in Germany and have German nationality. Neonates fulfilling at least one of the following criteria were excluded from the study: premature birth (maturity at <37 gestational weeks); low birth weight (<2500 g); congenital malformation; symptomatic intensive medical care during the neonatal period; immune-related diseases of the mother, such as autoimmune disorders; diabetes;

hepatitis B; long-term medication use; or abuse of drugs or alcohol. The current analysis is limited to children born in Wesel.

The MAAS study is an unselected, prospective population-based birth cohort study specifically designed to determine risk factors for the development of asthma and allergies. Recruitment took place in antenatal clinics of two hospitals, South Manchester University Hospitals NHS Foundation Trust (Wythenshawe) and Stepping Hill Hospital, between October 1995 and July 1997. During recruitment both parents completed a screening questionnaire on their history of asthma and allergic diseases and smoking habits and underwent skin prick tests. Based on parental allergic status children were assigned to high, medium or low risk groups. Families in the high risk group without pets were invited to participate in an intervention study. The intervention study involved stringent environmental controls to study the effects of allergen exposure on the development of asthma and allergies. The intervention group comprised 145 children, while the remainder of the cohort was treated as an observational cohort.

For the PIAMA birth cohort study, pregnant women were recruited in 1996-1997 during their second trimester of pregnancy from a series of communities in the North, West, and Centre of The Netherlands. Non-allergic pregnant women were invited to participate in a "natural history" study arm. Pregnant women identified as allergic through a validated screening questionnaire were primarily allocated to an intervention arm with a random subset allocated to the natural history arm. The intervention involved the use of mite-impermeable mattress and pillow covers.

Lung function measurements

In the BAMSE cohort, at eight years the children were invited to a clinical examination including lung function testing, in which 2,630 children participated. Peak expiratory flow was measured

using the normal-range Ferraris Peak Flow Meter ® (Ferraris Medical Limited, London, UK). The highest obtained PEF value from several successive attempts was used for analysis, provided that the child´s effort was coded as being maximal by the test leader, and that the two highest readings were reproducible (within 15% of each other). Maximum expiratory flow volume (MEFV) tests were performed using a spirometer (2200 Pulmonary Function Laboratory; Sensormedics, Anaheim, CA, USA). All children performed several MEFV measurements sitting, using a nose clip. The highest values of forced vital capacity (FVC) and forced expiratory volume in 1 sec (FEV_1) were extracted and used for analysis, provided that the child´s effort was coded as being maximal by the test leader, the MEFV curve passed visual quality inspection, and that the two highest readings were reproducible according to ATS/ERS criteria (Miller et al. 2005). Body weight and height were measured during clinical examination by trained test leaders. Body weight was measured with electronic calibrated equipment to the nearest 0.1 kg, while the children wore all clothes except outdoor clothes and thick sweaters. The children's height was measured to the nearest 0.1 cm.

In the GINI South cohort, at age six years, 762 children participated in a medical examination which included pulmonary function testing. From all participants, 1-5 forced expiration maneuvers following deep inspiration were recorded with a pneumotachograph from Jaeger (Viasys). Specifically, FVC, forced expiratory volume in one 0.5 seconds ($FEV_{0.5}$), in 0.75 seconds ($FEV_{0.75}$), and in one second (FEV_1), peak expiratory flow (PEF) and mid expiratory flows (FEF_{25}, FEF_{50}, FEF_{75}) were measured in sitting position after at least 15 minutes of rest, while wearing a nose clip, by trained personnel, in line with the ATS/ERS guidelines (Miller et al. 2005). For each child, the aim was to get at least three acceptable manoeuvres, however a maximum of 8 attempts were allowed. The data underwent visual inspection and acceptable

curves were assessed according to ATS/ERS guidelines. After the application of these quality controls, 546 PEF, 497 $FEV_{0.75}$, and 659 $FEV_{0.5}$ measurements were deemed acceptable for use and are included in this analysis. Body weight and height were measured during the medical examination and were performed by trained research staff using calibrated measuring equipment. Body weight was measured to the nearest gram and height to the nearest centimetre (no decimals). All anthropometric variables were measured while the child was wearing light clothing and no shoes**.**

In the GINI/LISA North cohort, at age six years, 987 children (875 GINI, 112 LISA,) participated in a medical examination which included pulmonary function testing. From all participants, 1-5 forced expiration maneuvers following deep inspiration were recorded with a pneumotachograph from Jaeger (Viasys). Specifically, FVC, $FEV_{0.5}$, $FEV_{0.75}$, FEV_1, PEF and mid expiratory flows (FEF_{25}, FEF_{50}, FEF_{75}) were measured in sitting position after at least 15 minutes of rest, while wearing a nose clip, by trained personnel, in line with the ATS/ERS guidelines (Miller et al. 2005). For each child, the aim was to get at least three acceptable manoeuvres, however a maximum of 8 attempts were allowed. The data underwent visual inspection and acceptable curves were assessed according to ATS/ERS guidelines. After the application of these quality controls, 781 (703 GINI, 78 LISA,) PEF, 859 (766 GINI, 93 LISA,) FEV0.75, and 968 (862 GINI, 106 LISA,) FEV0.5 measurements were deemed acceptable for use and are included in this analysis. Body weight and height were measured during the medical examination and were performed by trained research staff using calibrated measuring equipment. Body weight was measured to the nearest gram and height to the nearest centimetre (no decimals). All anthropometric variables were measured while the child was wearing light clothing and no shoes**.**

In the MAAS cohort, at age eight years all children were invited for a clinical follow up visit. The clinical follow up visit included amongst others a nurse administered a respiratory questionnaire based on the International Study of Asthma and Allergies in Childhood (ISAAC) and measurements of lung function. All children were asymptomatic at the time of assessment of lung function. Dynamic lung volumes were measured using a pneumotachograph based spirometer and incentive animation software (Jaeger, Germany), according to the American Thoracic Society guidelines. All measurements were made in a standing position without a nose-clip. The child was asked to inhale as deeply as possible i.e. to total lung capacity (TLC), then instructed to perform a forced expiration, through a mouthpiece, as hard and as fast as possible until no further gas could be exhaled i.e. to residual volume (RV). The test was repeated at intervals of 30 seconds until 3 technically acceptable traces were obtained and the highest $FEV_{1,}$ FVC and $FEV_{0.75}$ were recorded. Body weight and height were measured during the clinical follow up by trained research staff using calibrated measuring equipment.

In the PIAMA cohort, at age eight years, all children of allergic mothers and a random sample of children of non-allergic mothers were invited for a medical examination including pulmonary function testing (N = 1,552). In total, 1,132 children responded with a visit to one of the study hospitals. A Jaeger pneumotachograph (Viasys Healthcare, USA) was used for pulmonary function testing. The machines were calibrated on every medical examinations took place. FVC, FEV_1, PEF and mid expiratory flows (FEF_{25}, FEF_{50}, FEF_{75}) were measured in sitting position, while wearing a nose clip, by trained personnel, according to the ATS/ERS guidelines (Miller et al. 2005). For each child, at least three acceptable manoeuvres had to be obtained. Body weight and height were measured during the medical examination were performed by trained research staff using calibrated measuring equipment. Body weight was measured at the nearest 0.1kg and

height (cm) was measured at one decimal. All anthropometric variables were measured while the children were only wearing underwear.

Long-term air pollution exposure assessment

LUR models were developed for each pollution metric using all measurement sites, and in addition for background NO_2, using only regional and urban background sites. Overall model performance was evaluated by leave-one-out cross validation: each site was sequentially left out from the model while the included variables were left unchanged. Leave-one-out cross validation R^2 and root mean square errors of the models used for exposure estimation are presented in Supplemental Material, Table S1.

If values of predictor variables for the cohort addresses were outside the range of values for the monitoring sites, values were truncated to the minimum/maximum values at the monitoring sites.

Definition of allergic sensitization

Allergic sensitization was defined as specific IgE antibodies of ≥ 0.35 kUA/L for any allergen tested.

In the BAMSE cohort, blood samples collected at the age of 8 years were analyzed for allergen-specific serum IgE to a mix of common inhalant allergens Phadiatop® (birch, timothy, mugwort, cat, dog, horse, *Cladosporium herbarum* and house dust mite (*Dermatophagoides pteronyssinus*)) and a mix of common food allergens fx5 (cow's milk, egg white, soy bean, peanut, cod fish and wheat) with the ImmunoCAP System (Thermo Fisher/Phadia AB, Uppsala, Sweden).

In the GINI and LISA cohorts (North and South), blood samples collected at the age of 6 years were analyzed for allergen-specific serum IgE to common inhalant (birth, timothy, mugwort, cat, dog, *Cladosporium herbarum*, house dust mite (dermatophagoides pteronyssinus)) and food allergens (cow's milk, egg white, soy bean, peanut, cod fish, rye and wheat) with the CAP-RAST FEIA system (Pharmacia Diagnostics, Freiburg, Germany).

In the MAAS cohort, blood samples collected at the age of 8 years were analyzed for allergen-specific serum IgE to common inhalant (Gx1 mixed grasses, cat, dog, house mite) and food allergens (egg, milk, peanut) with the ImmunoCAP System (Thermo Fisher/Phadia AB, Uppsala, Sweden).

In the PIAMA cohort, blood samples collected at the age of 8 years were analyzed for allergen-specific serum IgE to common inhalant (birch, *Dactylis glomerata*, cat, dog, *Alternaria alternata*, house dust mite (*Dermatophagoides pteronyssinus*)) and food allergens (egg, milk) with the radioallergosorbent test-like method used at the Sanquin Laboratories (Amsterdam, The Netherlands).

References

Miller MR, Hankinson J, Brusasco V, Burgos F, Casaburi R, Coates A et al. 2005. Standardisation of spirometry. Eur Respir J 26:319-338.

Supplemental Material, Table S1. Performance of the Land-use regression models used for exposure estimation: leave-one-out cross-validation R^2 (R^2_{CV}) and root mean square error (RMSE) [μg/m^3].

	**BAMSE**		**GINI South**		**GINI/LISA North**		**MAAS**		**PIAMA**	
**Exposure**	$\mathbf{R^2_{CV}}$	**RMSE**	$\mathbf{R^2_{CV}}$	**RMSE**	$\mathbf{R^2_{CV}}$	**RMSE**	$\mathbf{R^2_{CV}}$	**RMSE**	$\mathbf{R^2_{CV}}$	**RMSE**
$\mathbf{NO_2}$	78%	3.5	67%	5.5	84%	4.3	75%	2.6	81%	5.1
$\mathbf{NO_x}$	79%	8.2	76%	9.4	81%	13.6	78%	5.6	82%	11.2
$\mathbf{PM_{2.5}}$	78%	0.8	62%	1.0	79%	0.9	21%	0.8	61%	1.2
$\mathbf{PM_{2.5}}$ **abs.**	85%	0.1	82%	0.2	95%	0.1	81%	0.1	89%	0.2
$\mathbf{PM_{10}}$	77%	3.3	75%	2.2	63%	2.0	75%	1.0	60%	2.3
$\mathbf{PM_{coarse}}$	65%	3.5	69%	1.6	57%	1.2	56%	1.0	38%	1.7

Supplemental Material, Table S2. Population characteristics of the baseline birth cohorts.

	BAMSE (N = 4089)		GINI South (N=2949)		GINI/LISA North (N = 3390)		MAAS (N = 1185)		PIAMA (N = 3963)	
**Variable**	**n/N**	**%**	**n/N**	**%**	**n/N**	**%**	**n/N**	**%**	**n/N**	**%**
Female sex	2024/4089	49.5	1361/2821	48.2**	1458/2989	48.8	543/1185	45.8	1908/3963	48.1*
Allergic mother	651/4032	16.2	1294/2930	44.2**	1076/3376	31.9**	683/1147	59.5	1237/3963	31.2**
Allergic father	665/4032	16.5**	1148/2905	39.5**	819/3333	24.6**	717/1138	63.0	1217/3957	30.8**
Native ethnicity/nationality [a]	2691/3398	79.2*	2949/2949	100.0	3390/3390	100.0	1059/1115	95.0	3485/3700	94.2**
High maternal SES [b]	1671/4062	41.1**	1632/2922	55.9**	981/3346	29.3**	NA		1331/3807	35.0**
High paternal SES [b]	1574/3982	39.5	1861/2875	64.7*	1112/3291	33.8**	204/1078	18.9	1493/3761	39.7**
Older siblings	1980/4088	48.4*	1181/2925	40.4	1810/3363	53.8	561/1073	52.3	1994/3937	50.6
Breastfeeding (≥12 wks)	3657/3879	94.3**	1475/2200	67.0	1362/2713	50.2**	502/1115	45.0**	1892/3896	48.6**
Mother smoked during pregnancy	527/4088	12.9**	313/2314	13.5	497/2816	17.6**	119/1025	11.6	696/3904	17.8**
Smoking at child's home										
Early life	855/4067	21.0	379/2296	16.5	843/2786	30.3**	438/1024	42.8	1129/3935	28.7**
Current [b]	597/3382	17.7	420/1940	21.6	817/2138	38.2*	366/999	36.6	548/3254	16.8
Use of natural gas for cooking										
Early life	471/4089	11.5	180/2297	7.8	123/2758	4.5	801/1030	77.8	3236/3911	82.7
Current [b]	235/3405	6.9	138/1936	7.1	74/2137	3.5	819/1030	79.5	2817/3601	78.2
Mold/dampness in child's home										
Early life	1034/4077	25.4	703/2295	30.6	542/2760	19.6	177/1030	17.2	1047/3702	28.3
Current [b]	332/3399	9.8	427/1931	22.1	310/2108	14.7**	149/1030	14.5	941/3238	29.1
Furry pets in home										
Early life	629/4089	15.4	365/2202	16.6	517/2686	19.2**	375/1028	36.5	1845/3937	46.9**
Current [b]	917/3403	26.9**	480/1942	24.7	606/2138	28.3	465/1029	45.2	1755/3210	54.7**
Day-care center attendance [c]	3205/3841	83.4**	165/2019	8.2	43/2402	1.8	741/1087	68.2	1034/3703	27.9
Study arm										
Intervention group	NA		1165/2949	39.5**	1087/3390	32.1**	133/1185	11.2	759/3941	19.3**
Birth weight [g]; mean ± std, N	3530 ± 558	4044	3412 ± 443	2212	3531 ± 476	2772	3462 ± 510	1132	3507 ± 546	3930

[a] BAMSE: Scandinavian, GINI/LISA: German; MAAS: Caucasian, PIAMA: Dutch. [b] SES=socio-economic status; defined by education for BAMSE, GINI/LISA and PIAMA and by income (>£ 30,000) in MAAS. [c]during 2nd year of life.

NA = not applicable/not available. Population characteristics of participants who were and who were not included in the current analysis were compared by means of chi-squared tests and t-tests (birth weight). * p-value < 0.1, ** p-value < 0.05

Supplemental Material, Table S3. Pearson correlations between long-term air pollution exposure estimates at the current and birth address for the BAMSE cohort.

	Birth address									Current address								
	**NO_2**	**NO_x**	**$PM_{2.5}$**	**$PM_{2.5}$ abs**	**PM_{10}**	**PM_{coarse}**	**NO_2 backgr.**	**Traffic intensity**	**Traffic load**	**NO_2**	**NO_x**	**$PM_{2.5}$**	**$PM_{2.5}$ abs**	**PM_{10}**	**PM_{coarse}**	**NO_2 backgr.**	**Traffic intensity**	**Traffic load**
**Birth address**																		
NO_2	1.00	0.96	0.75	0.93	0.62	0.63	0.79	0.54	0.42	0.59	0.56	0.40	0.56	0.39	0.39	0.44	0.29	0.27
NO_x		1.00	0.67	0.83	0.61	0.62	0.73	0.66	0.43	0.55	0.56	0.35	0.49	0.36	0.37	0.40	0.34	0.26
$PM_{2.5}$			1.00	0.89	0.57	0.58	0.76	0.38	0.40	0.46	0.41	0.58	0.56	0.34	0.35	0.45	0.21	0.26
$PM_{2.5}$ abs				1.00	0.67	0.68	0.81	0.44	0.48	0.56	0.50	0.49	0.62	0.41	0.42	0.47	0.24	0.31
PM_{10}					1.00	1.00	0.45	0.53	0.49	0.35	0.34	0.27	0.38	0.57	0.57	0.25	0.26	0.30
PM_{coarse}						1.00	0.45	0.55	0.51	0.36	0.35	0.27	0.38	0.56	0.57	0.25	0.27	0.31
NO_2 background							1.00	0.26	0.33	0.48	0.44	0.44	0.51	0.31	0.31	0.56	0.16	0.22
Traffic intensity								1.00	0.50	0.31	0.36	0.19	0.25	0.26	0.27	0.15	0.49	0.28
Traffic load									1.00	0.26	0.26	0.20	0.28	0.27	0.28	0.19	0.25	0.52
**Current address**																		
NO_2										1.00	0.96	0.71	0.91	0.54	0.56	0.79	0.56	0.45
NO_x											1.00	0.63	0.81	0.52	0.54	0.72	0.68	0.45
$PM_{2.5}$												1.00	0.88	0.50	0.51	0.74	0.33	0.40
$PM_{2.5}$ abs													1.00	0.60	0.62	0.81	0.41	0.51
PM_{10}														1.00	1.00	0.43	0.43	0.46
PM_{coarse}															1.00	0.44	0.45	0.48
NO_2 background																1.00	0.27	0.37
Traffic intensity																	1.00	0.44
Traffic load																		1.00

Supplemental Material, Table S4. Pearson correlations between estimated annual average air pollution levels at the current and birth address for the GINI South cohort.

	**Birth address**									**Current address**								
	**NO_2**	**NO_x**	**$PM_{2.5}$**	**$PM_{2.5}$ abs**	**PM_{10}**	**PM coarse**	**NO_2 backgr.**	**Traffic intensity**	**Traffic load**	**NO_2**	**NO_x**	**$PM_{2.5}$**	**$PM_{2.5}$ abs**	**PM_{10}**	**PM coarse**	**NO_2 backgr.**	**Traffic intensity**	**Traffic load**
**Birth address**																		
NO_2	1.00	0.95	0.46	0.74	0.68	0.92	0.70	0.31	0.56	0.56	0.50	0.17	0.25	0.29	0.47	0.45	0.15	0.19
NO_x		1.00	0.59	0.83	0.72	0.92	0.59	0.36	0.63	0.51	0.51	0.24	0.30	0.32	0.46	0.37	0.16	0.19
$PM_{2.5}$			1.00	0.62	0.53	0.47	0.17	0.26	0.45	0.15	0.20	0.48	0.17	0.20	0.13	0.03	0.05	0.06
$PM_{2.5}$ abs				1.00	0.72	0.81	0.33	0.35	0.66	0.32	0.34	0.24	0.39	0.28	0.35	0.14	0.16	0.18
PM_{10}					1.00	0.75	0.35	0.20	0.37	0.29	0.31	0.28	0.30	0.50	0.32	0.18	0.11	0.10
PM coarse						1.00	0.59	0.27	0.51	0.51	0.48	0.20	0.32	0.32	0.51	0.35	0.15	0.19
NO_2 background							1.00	0.13	0.26	0.44	0.36	0.02	0.10	0.17	0.31	0.65	0.07	0.12
Traffic intensity								1.00	0.40	0.12	0.13	0.06	0.13	0.10	0.12	0.02	0.41	0.12
Traffic load									1.00	0.21	0.21	0.11	0.19	0.10	0.19	0.08	0.14	0.26
**Current address**																		
NO_2										1.00	0.94	0.40	0.67	0.63	0.90	0.66	0.39	0.51
NO_x											1.00	0.52	0.77	0.66	0.90	0.53	0.46	0.58
$PM_{2.5}$												1.00	0.55	0.46	0.39	0.07	0.30	0.37
$PM_{2.5}$ abs													1.00	0.68	0.79	0.21	0.46	0.57
PM_{10}														1.00	0.71	0.30	0.26	0.30
PM coarse															1.00	0.49	0.35	0.45
NO_2 background																1.00	0.10	0.19
Traffic intensity																	1.00	0.74
Traffic load																		1.00

Supplemental Material, Table S5. Pearson correlations between estimated annual average air pollution levels at the current and birth address for the GINI/LISA North cohort.

	Birth address									Current address								
	**NO_2**	**NO_x**	**$PM_{2.5}$**	**$PM_{2.5}$ abs**	**PM_{10}**	**PM $_{coarse}$**	**NO_2 backgr.**	**Traffic intensity**	**Traffic load**	**NO_2**	**NO_x**	**$PM_{2.5}$**	**$PM_{2.5}$ abs**	**PM_{10}**	**PM $_{coarse}$**	**NO_2 backgr.**	**Traffic intensity**	**Traffic load**
**Birth address**																		
NO_2	1.00	0.98	0.73	0.76	0.74	0.62	0.66	0.16	0.46	0.67	0.66	0.53	0.49	0.51	0.40	0.53	0.10	0.29
NO_x		1.00	0.72	0.68	0.73	0.59	0.63	0.16	0.33	0.62	0.65	0.52	0.43	0.49	0.36	0.49	0.11	0.21
$PM_{2.5}$			1.00	0.71	0.83	0.67	0.61	0.08	0.25	0.56	0.59	0.86	0.49	0.61	0.51	0.53	0.05	0.14
$PM_{2.5}$ abs				1.00	0.82	0.68	0.56	0.31	0.69	0.56	0.54	0.54	0.64	0.61	0.51	0.47	0.17	0.34
PM_{10}					1.00	0.73	0.51	0.09	0.29	0.54	0.56	0.64	0.57	0.76	0.53	0.43	0.03	0.15
PM $_{coarse}$						1.00	0.53	0.11	0.29	0.45	0.46	0.53	0.47	0.55	0.75	0.45	0.07	0.17
NO_2 background							1.00	0.07	0.31	0.53	0.51	0.52	0.45	0.40	0.44	0.87	0.03	0.22
Traffic intensity								1.00	0.54	0.11	0.13	0.06	0.15	0.06	0.06	0.03	0.57	0.19
Traffic load									1.00	0.39	0.31	0.19	0.43	0.21	0.23	0.28	0.28	0.50
**Current address**																		
NO_2										1.00	0.96	0.68	0.79	0.68	0.56	0.63	0.15	0.61
NO_x											1.00	0.71	0.67	0.70	0.56	0.61	0.17	0.40
$PM_{2.5}$												1.00	0.64	0.77	0.63	0.59	0.08	0.22
$PM_{2.5}$ abs													1.00	0.78	0.63	0.54	0.25	0.74
PM_{10}														1.00	0.69	0.47	0.08	0.28
PM $_{coarse}$															1.00	0.49	0.10	0.25
NO_2 background																1.00	0.04	0.31
Traffic intensity																	1.00	0.34
Traffic load																		1.00

Supplemental Material, Table S6. Pearson correlations between estimated annual average air pollution levels at the current and birth address for the MAAS cohort.

	Birth address									Current address								
	**NO_2**	**NO_x**	**$PM_{2.5}$**	**$PM_{2.5}$ abs**	**PM_{10}**	**PM coarse**	**NO_2 backgr.**	**Traffic intensity**	**Traffic load**	**NO_2**	**NO_x**	**$PM_{2.5}$**	**$PM_{2.5}$ abs**	**PM_{10}**	**PM coarse**	**NO_2 backgr.**	**Traffic intensity**	**Traffic load**
**Birth address**																		
NO_2	1.00	0.66	0.40	0.41	0.39	0.35	0.53	0.18	0.11	0.66	0.44	0.35	0.29	0.24	0.24	0.45	0.04	0.03
NO_x		1.00	0.25	0.49	0.32	0.28	0.47	0.29	0.56	0.48	0.74	0.20	0.35	0.24	0.23	0.39	0.10	0.40
$PM_{2.5}$			1.00	0.18	0.27	0.26	0.16	0.00	-0.01	0.24	0.12	0.78	0.07	0.14	0.11	0.12	-0.03	-0.01
$PM_{2.5}$ abs				1.00	0.44	0.54	0.59	0.03	0.25	0.26	0.35	0.11	0.75	0.33	0.41	0.46	0.00	0.19
PM_{10}					1.00	0.66	0.29	0.04	0.14	0.27	0.23	0.24	0.37	0.52	0.33	0.25	-0.03	0.13
PM_{coarse}						1.00	0.27	0.00	0.03	0.26	0.21	0.23	0.45	0.29	0.64	0.23	-0.04	0.05
NO_2 background							1.00	-0.07	0.10	0.48	0.44	0.16	0.54	0.24	0.24	0.83	-0.03	0.09
Traffic intensity								1.00	0.09	0.10	0.13	-0.03	0.04	0.01	0.01	0.00	0.50	0.03
Traffic load									1.00	0.07	0.42	-0.02	0.17	0.14	0.04	0.05	0.03	0.71
**Current address**																		
NO_2										1.00	0.65	0.31	0.44	0.35	0.32	0.57	0.12	0.16
NO_x											1.00	0.21	0.52	0.39	0.32	0.49	0.19	0.59
$PM_{2.5}$												1.00	0.11	0.24	0.22	0.17	-0.03	-0.01
$PM_{2.5}$ abs													1.00	0.47	0.55	0.62	0.10	0.31
PM_{10}														1.00	0.62	0.29	-0.01	0.26
PM_{coarse}															1.00	0.26	-0.02	0.14
NO_2 background																1.00	-0.02	0.09
Traffic intensity																	1.00	0.09
Traffic load																		1.00

Supplemental Material, Table S7. Pearson correlations between estimated annual average air pollution levels at the current and birth address for the PIAMA cohort.

	Birth address									Current address								
	**NO_2**	**NO_x**	**$PM_{2.5}$**	**$PM_{2.5}$ abs**	**PM_{10}**	**PM $_{coarse}$**	**NO_2 backgr.**	**Traffic intensity**	**Traffic load**	**NO_2**	**NO_x**	**$PM_{2.5}$**	**$PM_{2.5}$ abs**	**PM_{10}**	**PM $_{coarse}$**	**NO_2 backgr.**	**Traffic intensity**	**Traffic load**
**Birth address**																		
NO_2	1.00	0.88	0.76	0.92	0.82	0.76	0.87	0.21	0.37	0.85	0.71	0.65	0.78	0.65	0.59	0.78	0.11	0.21
NO_x		1.00	0.74	0.89	0.88	0.76	0.66	0.25	0.42	0.72	0.71	0.59	0.69	0.62	0.56	0.61	0.13	0.20
$PM_{2.5}$			1.00	0.88	0.70	0.60	0.62	0.26	0.37	0.66	0.58	0.77	0.70	0.48	0.42	0.58	0.11	0.17
$PM_{2.5}$ abs				1.00	0.91	0.75	0.77	0.28	0.45	0.77	0.68	0.68	0.79	0.67	0.55	0.70	0.14	0.21
PM_{10}					1.00	0.81	0.69	0.24	0.47	0.64	0.61	0.48	0.67	0.71	0.58	0.61	0.09	0.19
PM $_{coarse}$						1.00	0.76	0.35	0.41	0.61	0.55	0.43	0.56	0.58	0.69	0.64	0.15	0.17
NO_2 background							1.00	0.07	0.24	0.79	0.60	0.57	0.72	0.62	0.63	0.88	0.04	0.13
Traffic intensity								1.00	0.47	0.09	0.09	0.09	0.10	0.07	0.11	0.01	0.54	0.23
Traffic load									1.00	0.23	0.20	0.16	0.22	0.20	0.17	0.18	0.20	0.39
**Current address**																		
NO_2										1.00	0.88	0.75	0.92	0.79	0.73	0.87	0.19	0.28
NO_x											1.00	0.71	0.88	0.85	0.75	0.67	0.25	0.34
$PM_{2.5}$												1.00	0.86	0.64	0.53	0.64	0.16	0.23
$PM_{2.5}$ abs													1.00	0.89	0.71	0.80	0.22	0.32
PM_{10}														1.00	0.77	0.70	0.14	0.31
PM $_{coarse}$															1.00	0.74	0.26	0.26
NO_2 background																1.00	0.03	0.15
Traffic intensity																	1.00	0.43
Traffic load																		1.00

Supplemental Material, Table S8. Pearson correlations between short-term exposures and estimated annual average air pollution levels at the birth and current address.

	BAMSE				GINI South		GINI/LISA North		MAAS		PIAMA			
**Short-term** / **Long-term**	**NO_2**	**NO_x**	**PM_{10}**	**$PM_{2.5}$**	**NO_2**	**PM_{10}**	**NO_2**	**PM_{10}**	**NO_2**	**PM_{10}**	**NO_2**	**NO_x**	**PM_{10}**	**BS**
**Birth address**														
NO_2	-0.02	-0.02	-0.00	-0.01	0.01	0.02	-0.03	-0.02	-0.03	-0.04	0.48	0.27	0.16	0.10
NO_x	-0.03	-0.03	-0.01	-0.01	0.01	0.01	-0.04	-0.02	-0.03	-0.05	0.37	0.20	0.12	0.07
$PM_{2.5}$	0.02	0.01	0.01	0.01	0.00	0.02	-0.02	-0.01	-0.03	-0.01	0.38	0.20	0.09	0.18
$PM_{2.5}$ abs	-0.01	-0.01	-0.00	0.00	-0.05	-0.05	0.02	-0.06	0.01	-0.00	0.44	0.25	0.16	0.11
PM_{10}	0.01	0.01	-0.02	0.00	-0.04	-0.05	0.01	-0.02	-0.03	0.01	0.32	0.18	0.14	0.03
PM_{coarse}	0.01	0.01	-0.02	0.00	-0.02	-0.01	0.01	-0.05	-0.04	-0.00	0.32	0.19	0.11	-0.00
NO_2 background	-0.01	-0.01	0.02	-0.01	0.02	0.03	-0.08	-0.02	0.01	-0.00	0.53	0.32	0.20	0.09
Traffic intensity nearest street	-0.01	-0.01	-0.00	0.00	0.00	-0.01	0.01	-0.03	0.01	-0.02	-0.04	-0.03	-0.05	-0.04
Traffic load major roads 100m buffer	0.02	0.01	-0.01	0.01	-0.06	-0.03	0.04	-0.03	-0.01	-0.05	0.06	0.02	-0.01	-0.03
**Current address**														
NO_2	-0.00	-0.01	0.03	0.02	0.01	0.00	-0.00	-0.04	-0.01	-0.04	0.53	0.31	0.20	0.11
NO_x	-0.01	-0.02	0.02	0.02	0.02	-0.00	-0.00	-0.05	-0.00	-0.01	0.41	0.24	0.16	0.09
$PM_{2.5}$	0.04	0.03	0.04	0.01	-0.02	-0.04	-0.03	-0.02	-0.04	-0.05	0.45	0.27	0.13	0.23
$PM_{2.5}$ abs	0.02	0.01	0.03	0.02	-0.00	-0.03	-0.04	-0.05	0.00	-0.04	0.50	0.30	0.19	0.14
PM_{10}	0.03	0.03	0.00	-0.00	-0.03	-0.03	-0.02	-0.03	0.00	-0.04	0.36	0.22	0.18	0.04
PM_{coarse}	0.03	0.03	0.00	-0.00	0.02	-0.01	-0.02	-0.04	-0.04	-0.03	0.37	0.23	0.17	0.02
NO_2 background	0.01	-0.00	0.05	0.01	0.01	0.00	-0.09	-0.02	0.05	-0.01	0.57	0.35	0.25	0.11
Traffic intensity nearest street	-0.01	-0.01	0.01	0.02	-0.03	-0.03	-0.04	-0.00	-0.01	-0.01	-0.01	-0.00	-0.02	-0.02
Traffic load major roads 100m buffer	0.02	0.02	0.02	0.01	-0.05	-0.05	-0.01	-0.02	-0.03	-0.07	0.04	0.01	0.01	-0.02

BS = black smoke

Supplemental Material, Table S9. Crude and adjusted associations [a] of annual average levels of air pollution and traffic indicators with FVC: results from random-effects meta-analyses.

	Crude [b,c]		Adjusted [d,e]	
**Exposure**	**% diff. (95% CI)**	**I^2 (p_{het})**	**% diff. (95% CI)**	**I^2 (p_{het})**
**Birth address**				
NO_2	-0.87 (-3.30, 1.62)	90.7 (0.0000)	-1.19 (-3.57, 1.24)	79.5 (0.0076)
NO_x	-0.46 (-2.05, 1.16)	78.4 (0.0097)	-0.62 (-2.12, 0.90)	63.1 (0.0664)
$PM_{2.5}$ absorbance	-1.77 (-5.84, 2.47)	81.8 (0.0041)	-3.02 (-6.02, 0.08)	43.8 (0.1688)
$PM_{2.5}$	-2.95 (-11.28, 6.15)	88.1 (0.0002)	-3.39 (-11.18, 5.09)	83.7 (0.0021)
PM_{10}	-1.16 (-6.16, 4.09)	70.6 (0.0334)	-1.94 (-6.09, 2.39)	56.6 (0.1000)
PM_{coarse}	-2.66 (-8.21, 3.23)	90.0 (0.0000)	-3.33 (-8.57, 2.22)	87.7 (0.0003)
Traffic intensity nearest street	0.20 (-0.25, 0.65)	0.0 (0.9079)	0.25 (-0.22, 0.72)	0.0 (0.9904)
Traffic load major roads 100m buffer	0.22 (-0.42, 0.87)	0.0 (0.7022)	0.05 (-0.64, 0.74)	0.0 (0.5838)
**Current address**				
NO_2	-1.77 (-4.11, 0.63)	88.5 (0.0002)	-2.14 (-4.20, -0.04)	79.8 (0.0070)
NO_x	-1.57 (-3.49, 0.38)	82.7 (0.0031)	-1.93 (-3.42, -0.41)	64.4 (0.0601)
$PM_{2.5}$ absorbance	-4.13 (-9.04, 1.05)	85.6 (0.0010)	-5.57 (-10.00, -0.92)	76.8 (0.0134)
$PM_{2.5}$	-6.63 (-17.08, 5.13)	91.5 (0.0000)	-8.83 (-20.47, 4.52)	91.9 (0.0000)
PM_{10}	-5.15 (-13.65, 4.18)	87.4 (0.0004)	-6.22 (-14.72, 3.13)	85.1 (0.0012)
PM_{coarse}	-4.02 (-11.25, 3.81)	92.3 (0.0000)	-4.81 (-11.40, 2.28)	88.8 (0.0001)
Traffic intensity nearest street	-0.26 (-0.74, 0.23)	0.0 (0.9526)	-0.34 (-0.85, 0.18)	0.0 (0.7590)
Traffic load major roads 100m buffer	0.09 (-0.62, 0.81)	0.0 (0.9708)	-0.15 (-0.91, 0.62)	0.0 (0.8453)

[a] Associations are expressed as percent change with 95% confidence intervals, I^2 and p-value of test for heterogeneity of effect estimates between cohorts and presented for the following increments in exposure: 10 µg/m³ for NO_2, 20 µg/m³ for NO_x, 1 unit for $PM_{2.5}$ absorbance, 5 µg/m³ for $PM_{2.5}$, 10 µg/m³ for PM_{10}, 5 µg/m³ for PM_{coarse}, 5,000 veh/day for traffic intensity on the nearest street; and 4,000 veh-km/day for traffic load on major roads within a 100 m buffer.

[b] Adjusted for age, sex, height and weight all participants; associations with traffic intensity and traffic load were additionally adjusted for background NO_2 concentrations.

[c]N = 3,739 for birth address and N = 3,622 for current address.

[d]Crude model additionally adjusted for recent respiratory infections, ethnicity/nationality, parental education, allergic mother, allergic father, breastfeeding, mother smoking during pregnancy, smoking at home, mold/dampness at home, furry pets at home, and study region (BAMSE only).

[e]N = 3,457 for birth address and N = 3,233 for current address.

Supplemental Material, Table S10. Crude and adjusted associations [a] of annual average air pollution levels and traffic indicators with PEF: results from random-effects meta-analyses.

	Crude [b,c]		Adjusted [d,e]	
**Exposure**	**% diff. (95% CI)**	**I^2 (p_{het})**	**% diff. (95% CI)**	**I^2 (p_{het})**
**Birth address**				
NO_2	-0.25 (-0.96, 0.47)	0.0 (0.9787)	-0.51 (-1.49, 0.49)	0.0 (0.9782)
NO_x	-0.22 (-0.90, 0.45)	0.0 (0.9369)	-0.23 (-1.13, 0.69)	0.0 (0.8234)
$PM_{2.5}$ absorbance	-0.16 (-2.09, 1.80)	0.0 (0.9182)	-0.41 (-2.87, 2.11)	0.0 (0.8786)
$PM_{2.5}$	-0.69 (-2.51, 1.15)	0.0 (0.7292)	-0.88 (-3.21, 1.52)	0.0 (0.9136)
PM_{10}	-0.03 (-1.30, 1.25)	0.0 (0.9590)	0.10 (-1.35, 1.57)	0.0 (0.9672)
PM_{coarse}	-0.11 (-0.94, 0.73)	0.0 (0.8255)	-0.07 (-1.03, 0.89)	0.0 (0.8029)
Traffic intensity nearest street	-0.09 (-0.57, 0.40)	0.0 (0.5186)	0.03 (-0.46, 0.53)	0.0 (0.7651)
Traffic load major roads 100m buffer	0.39 (-0.61, 1.39)	0.0 (0.6208)	0.20 (-0.84, 1.25)	0.0 (0.7559)
**Current address**				
NO_2	-0.89 (-1.66, -0.11)	0.0 (0.9850)	-1.04 (-1.94, -0.13)	0.0 (0.9879)
NO_x	-0.80 (-1.55, -0.04)	0.0 (0.9551)	-0.82 (-1.69, 0.05)	0.0 (0.9565)
$PM_{2.5}$ absorbance	-1.54 (-3.62, 0.59)	0.0 (0.5209)	-2.16 (-4.55, 0.29)	0.0 (0.4496)
$PM_{2.5}$	-1.86 (-3.63, -0.06)	0.0 (0.4901)	-2.07 (-4.05, -0.04)	0.0 (0.7845)
PM_{10}	-0.87 (-2.23, 0.52)	0.0 (0.8072)	-1.48 (-2.96, 0.03)	0.0 (0.6650)
PM_{coarse}	-1.46 (-4.52, 1.69)	43.6 (0.1498)	-2.26 (-6.26, 1.91)	60.1 (0.0571)
Traffic intensity nearest street	0.14 (-0.41, 0.69)	0.0 (0.5035)	0.08 (-0.49, 0.66)	0.0 (0.6417)
Traffic load major roads 100m buffer	0.73 (-0.45, 1.93)	0.0 (0.5459)	0.47 (-0.76, 1.71)	0.0 (0.6044)

[a] Associations are expressed as percent change with 95% confidence intervals, I^2 and p-value of test for heterogeneity of effect estimates between cohorts and presented for the following increments in exposure: 10 µg/m³ for NO_2,20 µg/m³ for NO_x, 1 unit for $PM_{2.5}$ absorbance, 5 µg/m³ for $PM_{2.5}$, 10 µg/m³ for PM_{10}, 5 µg/m³ for PM_{coarse}, 5,000 veh/day for traffic intensity on the nearest street; and 4,000 veh-km/day for traffic load on major roads within a 100 m buffer.

[b] Adjusted for age, sex, height and weight all participants; associations with traffic intensity and traffic load were additionally adjusted for background NO_2 concentrations.

[c] N = 4,916 for birth address and N = 4,816 for current address

[d] Crude model additionally adjusted for recent respiratory infections, ethnicity/nationality, parental education, allergic mother, allergic father, breastfeeding, mother smoking during pregnancy, smoking at home, mold/dampness at home, furry pets at home, and study region (BAMSE only).

[d] N = 4,546 for birth address and N = 4,367 for current address

Supplemental Material, Table S11. Adjusted [a] associations b of average air pollution levels on the seven days preceding the lung function measurements with lung function: results from random-effects meta-analyses.

Exposure	% diff. (95% CI)	I^2 (p_{het})
**FEV_1 [c]**		
NO_2	-0.28 (-0.68, 0.13)	0.0 (0.7114)
PM_{10}	-0.18 (-0.65, 0.30)	39.4 (0.1587)
**FVC [d]**		
NO_2	-0.70 (-1.66, 0.28)	73.3 (0.0236)
PM_{10}	-0.20 (-0.89, 0.49)	51.3 (0.1286)
**PEF [e]**		
NO_2	-0.12 (-0.73, 0.49)	0.0 (0.7983)
PM_{10}	-0.22 (-0.89, 0.46)	49.4 (0.1154)

[a]Adjusted for age, sex, height and weight all participants, recent respiratory infections, ethnicity/nationality, parental education, allergic mother, allergic father, breastfeeding, mother smoking during pregnancy, smoking at home, mold/dampness at home, furry pets at home, and study region (BAMSE only).

[b]Associations are expressed as percent change with 95% confidence intervals, I^2 and p-value of test for heterogeneity of effect estimates between cohorts and presented for increments of
10 µg/m³.

[c] N = 4,919

[d] N = 3,466

[e] N = 4,454

Supplemental Material, Table S12. Adjusted [a] associations [b] of annual average air pollution levels and traffic indicators at the current address with FEV_1 for asthmatic and non-asthmatic children separately: results from random-effects meta-analyses.

Exposure	Asthmatics [c]		Non-asthmatics [d]		p-value (interaction)
	% diff. (95% CI)	I^2 (p_{het})	% diff. (95% CI)	I^2 (p_{het})	
NO_2	-0.14 (-5.67, 5.72)	45.0 (0.1219)	-1.15 (-1.89, -0.41)	0.0 (0.6375)	0.862
NO_x	-0.52 (-6.24, 5.56)	49.1 (0.0967)	-0.95 (-1.67, -0.22)	0.0 (0.9624)	0.407
$PM_{2.5}$ absorbance	-3.24 (-13.9, 8.79)	35.4 (0.1853)	-2.78 (-4.64, -0.89)	0.0 (0.5240)	0.908
$PM_{2.5}$	0.09 (-5.93, 6.49)	0.0 (0.4176)	-2.44 (-4.26, -0.58)	0.0 (0.4869)	0.463
PM_{10}	-6.83 (-19.8, 8.21)	26.1 (0.2478)	-0.90 (-2.92, 1.16)	12.1 (0.3363)	0.904
PM_{coarse}	-8.35 (-19.8, 4.74)	51.5 (0.0830)	-1.14 (-3.34, 1.11)	36.1 (0.1805)	0.887
Traffic intensity nearest street	1.10 (-0.62, 2.84)	0.0 (0.5197)	-0.25 (-0.69, 0.18)	0.0 (0.8308)	0.302
Traffic load major roads 100m buffer	0.97 (-1.75, 3.77)	0.0 (0.6972)	-0.07 (-0.78, 0.66)	0.0 (0.7967)	0.304

[a]Adjusted for age, sex, height and weight all participants, recent respiratory infections, ethnicity/nationality, parental education, allergic mother, allergic father, breastfeeding, mother smoking during pregnancy, smoking at home, mold/dampness at home, furry pets at home, and study region (BAMSE only); associations with traffic intensity and traffic load were additionally adjusted for background NO_2 concentrations.

[b]Associations are expressed as percent change with 95% confidence intervals, I^2 and p-value of test for heterogeneity of effect estimates between cohorts and presented for the following increments in exposure: 10 µg/m³ for NO_2,20 µg/m³ for NO_x, 1 unit for $PM_{2.5}$ absorbance, 5 µg/m³ for $PM_{2.5}$, 10 µg/m³ for PM_{10}, 5 µg/m³ for PM_{coarse}, 5,000 veh/day for traffic intensity on the nearest street; and 4,000 veh-km/day for traffic load on major roads within a 100 m buffer.

[c] N = 432

[d] N = 4,212

Supplemental Material, Table S13. Adjusted [a] associations [b] of annual average air pollution levels and traffic indicators at the current address with FEV_1 for sensitized and non-sensitized children separately: results from random-effects meta-analyses.

	Sensitized [c]		Non-sensitized [d]		p-value
**Exposure**	**% diff. (95% CI)**	**I^2 (p_{het})**	**% diff. (95% CI)**	**I^2 (p_{het})**	**(interaction)**
NO_2	-0.92 (-2.16, 0.33)	0.0 (0.7915)	-0.84 (-1.96, 0.28)	18.5 (0.2971)	0.822
NO_x	-1.15 (-2.34, 0.06)	0.0 (0.7837)	-0.72 (-1.66, 0.22)	0.0 (0.4676)	0.771
$PM_{2.5}$ absorbance	-0.43 (-3.73, 2.99)	0.0 (0.9277)	-2.95 (-6.16, 0.38)	41.9 (0.1424)	0.265
$PM_{2.5}$	-0.22 (-3.27, 2.93)	0.0 (0.9028)	-3.48 (-5.73, -1.18)	0.0 (0.8326)	0.088
PM_{10}	1.24 (-1.10, 3.64)	0.0 (0.8411)	-1.35 (-3.15, 0.48)	0.0 (0.7841)	0.121
PM_{coarse}	0.72 (-0.82, 2.28)	0.0 (0.8489)	-1.03 (-3.42, 1.43)	22.3 (0.2724)	0.225
Traffic intensity nearest street	-0.49 (-1.48, 0.52)	22.7 (0.2696)	-0.19 (-0.71, 0.33)	0.0 (0.7026)	0.375
Traffic load major roads 100m buffer	-0.12 (-1.40, 1.18)	5.5 (0.3756)	-0.20 (-1.28, 0.90)	0.0 (0.5439)	0.175

[a]Adjusted for age, sex, height and weight all participants, recent respiratory infections, ethnicity/nationality, parental education, allergic mother, allergic father, breastfeeding, mother smoking during pregnancy, smoking at home, mold/dampness at home, furry pets at home, and study region (BAMSE only); associations with traffic intensity and traffic load were additionally adjusted for background NO_2 concentrations.

[b]Associations are expressed as percent change with 95% confidence intervals, I^2 and p-value of test for heterogeneity of effect estimates between cohorts andpresented for the following increments in exposure: 10 µg/m³ for NO_2,20 µg/m³ for NO_x, 1 unit for $PM_{2.5}$ absorbance, 5 µg/m³ for $PM_{2.5}$, 10 µg/m³ for PM_{10}, 5 µg/m³ for PM_{coarse}, 5,000 veh/day for traffic intensity on the nearest street; and 4,000 veh-km/day for traffic load on major roads within a 100 m buffer.

[c] N = 1,532

[d] N = 2,545

Supplemental Material, Table S14. Adjusted [a] associations [b] of annual average levels of air pollution and traffic indicators at the current address with FEV_1 for boys and girls separately: results from random-effects meta-analyses.

Exposure	Girls [c] % diff. (95% CI)	Girls [c] I^2 (p_{het})	Boys [d] % diff. (95% CI)	Boys [d] I^2 (p_{het})	p-value (interaction)
NO_2	-0.95 (-1.96, 0.07)	0.0 (0.9168)	-0.88 (-1.99, 0.24)	8.2 (0.3599)	0.879
NO_x	-0.81 (-1.80, 0.19)	0.0 (0.8016)	-0.80 (-1.81, 0.22)	0.0 (0.6942)	0.794
$PM_{2.5}$ absorbance	-1.35 (-4.38, 1.77)	17.8 (0.3014)	-2.80 (-5.34, -0.19)	0.0 (0.6925)	0.673
$PM_{2.5}$	-1.97 (-4.38, 0.51)	0.0 (0.7063)	-3.99 (-8.05, 0.24)	34.5 (0.1914)	0.658
PM_{10}	-1.51 (-3.36, 0.38)	0.0 (0.5091)	-0.20 (-3.30, 3.01)	17.9 (0.3004)	0.402
PM_{coarse}	-0.76 (-2.00, 0.51)	0.0 (0.9850)	-1.87 (-6.08, 2.51)	66.4 (0.0180)	0.107
Traffic intensity nearest street	-0.13 (-0.94, 0.69)	24.6 (0.2574)	-0.29 (-0.85, 0.28)	0.0 (0.9043)	0.931
Traffic load major roads 100m buffer	0.41 (-1.37, 2.23)	24.0 (0.2614)	-0.16 (-1.00, 0.69)	0.0 (0.9771)	0.664

[a]Adjusted for age, height and weight all participants, recent respiratory infections, ethnicity/nationality, parental education, allergic mother, allergic father, breastfeeding, mother smoking during pregnancy, smoking at home, mold/dampness at home, furry pets at home, and study region (BAMSE only); associations with traffic intensity and traffic load were additionally adjusted for background NO_2 concentrations.

[b]Associations are expressed as percent change with 95% confidence intervals, I^2 and p-value of test for heterogeneity of effect estimates between cohorts and presented for the following increments in exposure: 10 µg/m³ for NO_2, 20 µg/m³ for NO_x, 1 unit for $PM_{2.5}$ absorbance, 5 µg/m³ for $PM_{2.5}$, 10 µg/m³ for PM_{10}, 5 µg/m³ for PM_{coarse}, 5,000 veh/day for traffic intensity on the nearest street; and 4,000 veh-km/day for traffic load on major roads within a 100 m buffer.

[c] N = 2,382

[d] N = 2,357

Supplemental Material, Table S15. Adjusted [a] associations [b] of annual average levels of air pollution and traffic indicators at the current address with FEV_1 for children with and without allergic parents separately: results from random-effects meta-analyses.

	**Allergic parents [c]**		**Non-allergic parents [d]**		**p-value**
**Exposure**	**% diff. (95% CI)**	**I^2 (p_{het})**	**% diff. (95% CI)**	**I^2 (p_{het})**	**(interaction)**
NO_2	-0.53 (-1.61, 0.57)	11.0 (0.3430)	-1.42 (-2.51, -0.32)	0.0 (0.7132)	0.398
NO_x	-0.51 (-1.50, 0.48)	0.0 (0.4636)	-1.08 (-2.10, -0.06)	0.0 (0.7347)	0.338
$PM_{2.5}$ absorbance	-1.72 (-4.08, 0.70)	0.0 (0.8723)	-3.10 (-6.22, 0.12)	7.2 (0.3658)	0.122
$PM_{2.5}$	-2.33 (-5.10, 0.52)	0.0 (0.8116)	-2.13 (-4.37, 0.17)	0.0 (0.4263)	0.092
PM_{10}	-1.31 (-3.52, 0.96)	0.0 (0.5718)	0.27 (-1.47, 2.05)	0.0 (0.7988)	0.087
PM_{coarse}	-0.70 (-3.06, 1.73)	25.0 (0.2550)	-1.61 (-5.62, 2.57)	35.8 (0.1829)	0.174
Traffic intensity nearest street	-0.02 (-0.69, 0.66)	0.0 (0.5292)	-0.37[e] (0.91, 0.18)	0.0 (0.5849)	0.700
Traffic load major roads 100m buffer	-0.06 (-1.27, 1.17)	0.0 (0.7492)	-0.02 (-0.95, 0.91)	0.0 (0.5456)	0.289

[a]Adjusted for age, sex, height and weight all participants, recent respiratory infections, ethnicity/nationality, parental education, breastfeeding, mother smoking during pregnancy, smoking at home, mold/dampness at home, furry pets at home, and study region (BAMSE only); associations with traffic intensity and traffic load were additionally adjusted for background NO_2 concentrations.

[b]Associations are expressed as percent change with 95% confidence intervals, I^2 and p-value of test for heterogeneity of effect estimates between cohorts and presented for the following increments in exposure: 10 µg/m³ for NO_2, 20 µg/m³ for NO_x, 1 unit for $PM_{2.5}$ absorbance, 5 µg/m³ for $PM_{2.5}$, 10 µg/m³ for PM_{10}, 5 µg/m³ for PM_{coarse}, 5,000 veh/day for traffic intensity on the nearest street; and 4,000 veh-km/day for traffic load on major roads within a 100 m buffer.

[c] N = 2,606

[d] N = 2,059

[e] N = 1,969, model did not converge for MAAS.

Supplemental Material, Table S16. Adjusted [a] associations [b] of annual average levels of air pollution and traffic indicators at the current address with FEV_1 stratified by moving between birth and lung function measurements: results from random-effects meta-analyses.

	Non-movers [c]		Movers [d]		p-value
**Exposure**	**% diff.(95% CI)**	**I^2 (p_{het})**	**% diff.(95% CI)**	**I^2 (p_{het})**	**(interaction)**
NO_2	-0.12 (-1.64, 1.42)	33.9 (0.1950)	-1.23 (-2.22, -0.23)	0.0 (0.5435)	0.449
NO_x	-0.22 (-1.31, 0.87)	0.0 (0.4317)	-1.29 (-2.50, -0.07)	18.5 (0.2947)	0.487
$PM_{2.5}$ absorbance	-1.14 (-3.86, 1.65)	0.0 (0.6555)	-2.90 (-5.42, -0.31)	0.0 (0.6755)	0.503
$PM_{2.5}$	-2.66 (-5.48, 0.24)	0.0 (0.9349)	-4.05 (-9.32, 1.52)	52.0 (0.0801)	0.560
PM_{10}	0.30 (-2.08, 2.74)	0.0 (0.8667)	-4.27 (-9.28, 1.02)	56.4 (0.0568)	0.603
PM_{coarse}	0.32 (-1.30, 1.96)	0.0 (0.5959)	-3.17 (-7.19, 1.03)	59.3 (0.0433)	0.851
Traffic intensity nearest street	-0.15 (-0.85, 0.56)	0.0 (0.9584)	-0.12 (-0.66, 0.43)	0.0 (0.7784)	0.637
Traffic load major roads 100m buffer	0.12 (-0.93, 1.17)	0.0 (0.8897)	0.13 (-0.82, 1.09)	0.0 (0.7467)	0.822

[a]Adjusted for age, sex, height and weight all participants, recent respiratory infections, ethnicity/nationality, parental education, allergic mother, allergic father, breastfeeding, mother smoking during pregnancy, smoking at home, mold/dampness at home, furry pets at home, and study region (BAMSE only); associations with traffic intensity and traffic load were additionally adjusted for background NO_2 concentrations.

[b]Associations are expressed as percent change with 95% confidence intervals, I^2 and p-value of test for heterogeneity of effect estimates between cohorts and presented for the following increments in exposure: 10 µg/m³ for NO_2,20 µg/m³ for NO_x, 1 unit for $PM_{2.5}$ absorbance, 5 µg/m³ for $PM_{2.5}$, 10 µg/m³ for PM_{10}, 5 µg/m³ for PM_{coarse}, 5,000 veh/day for traffic intensity on the nearest street; and 4,000 veh-km/day for traffic load on major roads within a 100 m buffer.

[c] N = 2,251

[d] N = 2,450

Supplemental Material, Table S17. Adjusted [a] associations [b] of back-extrapolated annual average levels of NO_x, NO_2, and PM_{10} at the birth address with lung function: results from random-effects meta-analyses.

	FEV_1 [c]		FVC [d]		PEF [e]	
**Exposure**	**% diff. (95% CI)**	**I^2 (p_{het})**	**% diff. (95% CI)**	**I^2 (p_{het})**	**% diff. (95% CI)**	**I^2 (p_{het})**
**Difference method**						
NO_2	-0.54 (-1.25, 0.17)	0.0 (0.6942)	-0.86 (-3.28, 1.62)	81.8 (0.0041)	-0.49 (-1.46, 0.50)	0.0 (0.9635)
NO_x	0.01 (-0.65, 0.68)	0.0 (0.7006)	-0.18 (-1.89, 1.56)	78.2 (0.0102)	-0.13 (-1.03, 0.78)	0.0 (0.7890)
PM_{10}	0.74 (-0.48, 1.97)	0.0 (0.9349)	-1.50 (-4.97, 2.10)	71.1 (0.0313)	0.81 (-0.90, 2.54)	0.0 (0.3583)
**Ratio method**						
NO_2	-0.34 (-0.86, 0.18)	0.0 (0.5906)	-0.73 (-2.51, 1.08)	85.5 (0.0010)	-0.33 (-1.00, 0.35)	0.0 (0.9659)
NO_x	0.04 (-0.35, 0.43)	0.0 (0.7951)	-0.24 (-1.22, 0.76)	76.6 (0.0138)	-0.07 (-0.55, 0.40)	0.0 (0.8161)
PM_{10}	0.50 (-0.52, 1.53)	0.0 (0.8735)	-1.35 (-4.22, 1.61)	71.1 (0.0313)	0.46 (-0.72, 1.65)	0.0 (0.5300)

[a]Adjusted for age, sex, height and weight all participants, recent respiratory infections, ethnicity/nationality, parental education, allergic mother, allergic father, breastfeeding, mother smoking during pregnancy, smoking at home, mold/dampness at home, furry pets at home, and study region (BAMSE only); associations with traffic intensity and traffic load were additionally adjusted for background NO_2 concentration.

[b]Associations are expressed as percent change with 95% confidence intervals, I^2 and p-value of test for heterogeneity of effect estimates between cohorts and presented for the following increments in exposure: 10 µg/m³ for NO_2,20 µg/m³ for NO_x, and 10 µg/m³ for PM_{10}.

[c] N = 4,887

[d] N = 3,457

[e] N = 4,546

Supplemental Material, Table S18. Adjusted [a] associations [b] of annual average levels of air pollution at the current address with lung function from two-pollutant models with NO_2 and $PM_{2.5}$: results from random-effects meta-analyses.

		Single-pollutant	Two-pollutant
**Outcome**	**Exposure**	**% diff. (95% CI)**	**% diff. (95% CI)**
**FEV_1**	NO_2	-0.98 (-1.70, -0.26)	-0.26 (-1.25, 0.73)
	$PM_{2.5}$	-2.49 (-4.57, -0.36)	-1.68 (-4.07, 0.76)
**FVC**	NO_2	-2.14 (-4.20 , -0.04)	-1.43 (-2.76, -0.09)
	$PM_{2.5}$	-8.83 (-20.47 , 4.52)	-3.67 (-8.70, 1.65)
**PEF**	NO_2	-1.04 (-1.94, -0.13)	-0.57 (-1.99, 0.86)
	$PM_{2.5}$	-2.07 (-4.05, -0.04)	-1.63 (-5.82, 2.75)

[a]Associations are expressed as percent change with 95% confidence intervals, I^2 and p-value of test for heterogeneity of effect estimates between cohorts and presented for the following increments in exposure: 10 µg/m³ for NO_2 and 5 µg/m³ for $PM_{2.5}$.

[b]Adjusted for age, sex, height and weight, recent respiratory infections, ethnicity/nationality, parental education, allergic mother, allergic father, breastfeeding, mother smoking during pregnancy, smoking at home, mold/dampness at home, furry pets at home, and study region (BAMSE only).

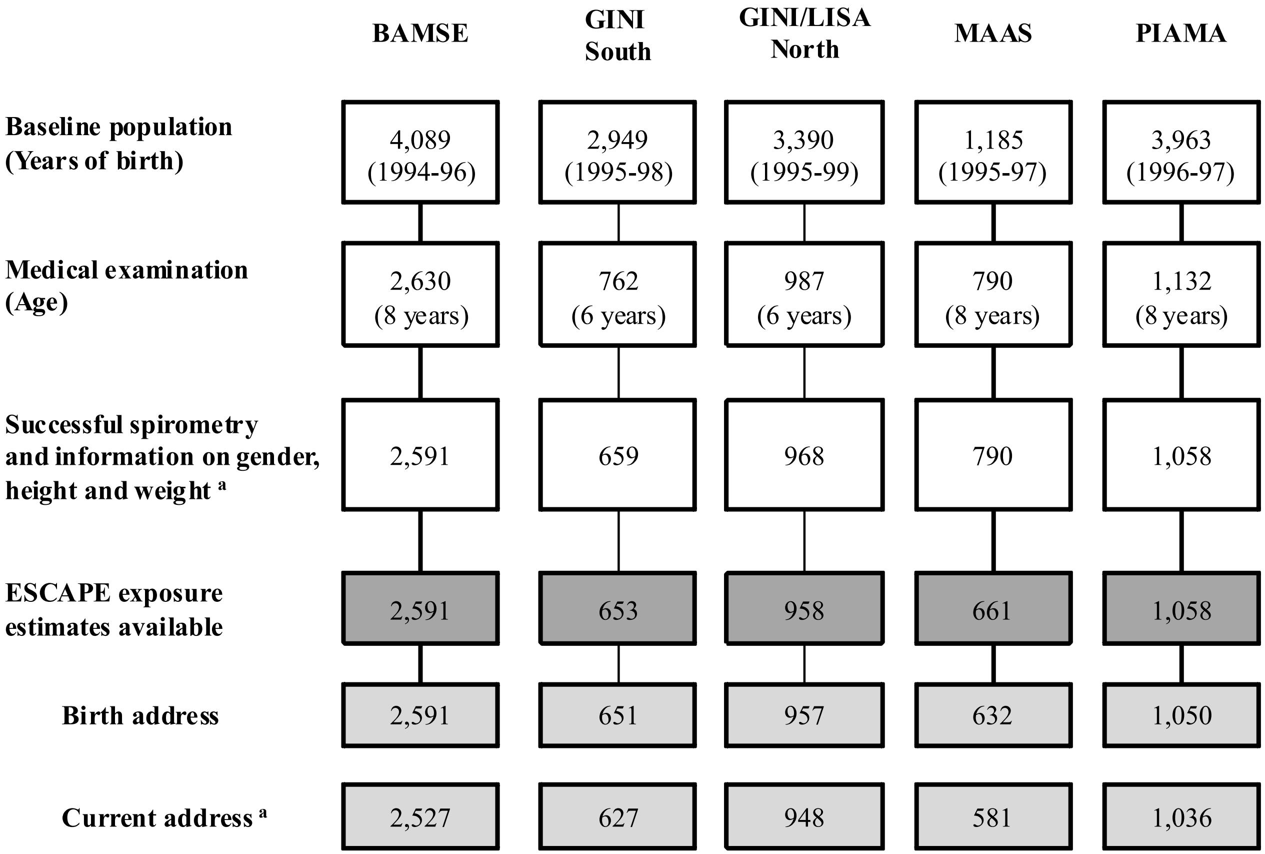

Supplemental Material, Figure S1. Study population. [a]At time of lung function measurements.

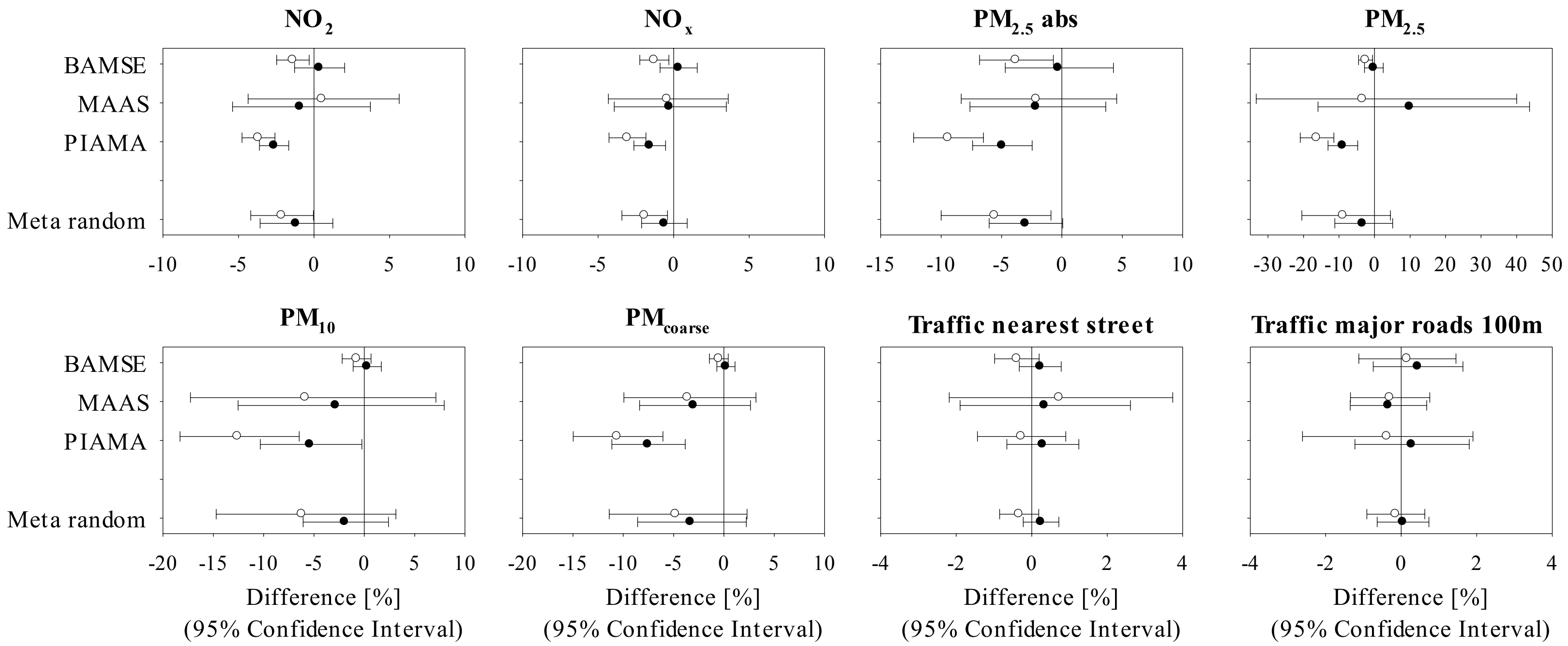

Supplemental Material, Figure S2. Forest plots of adjusted center-specific and combined associations of annual average air pollution levels and traffic indicators with FVC. Associations with exposures at birth address are represented by black dots, associations with exposures at current address by white dots. Estimates are adjusted for age, sex, height and weight, recent respiratory infections, ethnicity/nationality, parental education, allergic mother, allergic father, breastfeeding, mother smoking during pregnancy, smoking at home, mold/dampness at home, furry pets at home, and study region (BAMSE only); associations with traffic intensity and traffic load were additionally adjusted for background NO_2 concentrations. Associations are presented for the following increments in exposure: 10 µg/m³ for NO_2,20 µg/m³ for NO_x, 1 unit for $PM_{2.5}$ absorbance, 5 µg/m³ for $PM_{2.5}$, 10 µg/m³ for PM_{10}, 5 µg/m³ for PM_{coarse}, 5,000 veh·day^{-1}·m for traffic intensity on the nearest street; and 4,000 veh-km/day for traffic load on major roads within a 100 m buffer.

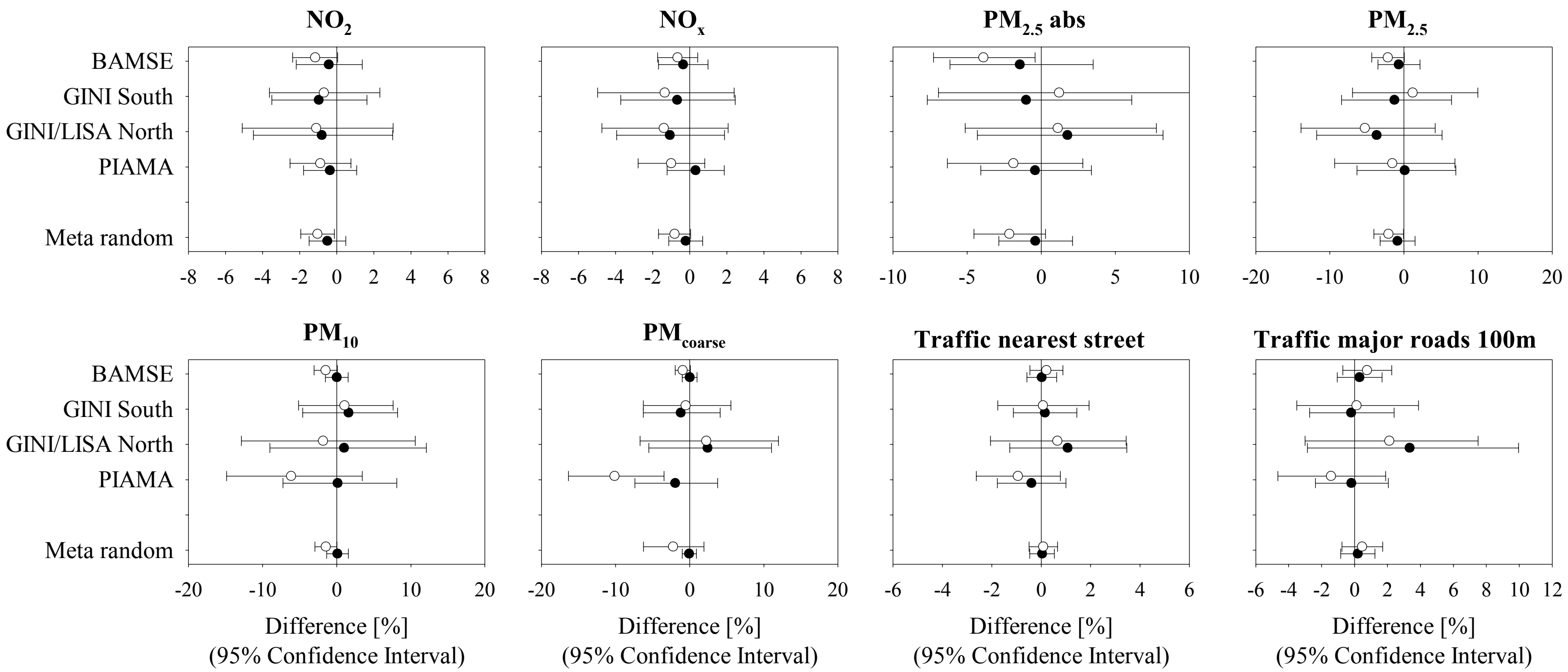

Supplemental Material, Figure S3. Adjusted center-specific and combined associations of annual average air pollution levels and traffic indicators with PEF. Associations with exposures at birth address are represented by black dots, associations with exposures at current address by white dots. Estimates are adjusted for age, sex, height and weight, recent respiratory infections, ethnicity/nationality, parental education, allergic mother, allergic father, breastfeeding, mother smoking during pregnancy, smoking at home, mold/dampness at home, furry pets at home, and study region (BAMSE only); associations with traffic intensity and traffic load were additionally adjusted for background NO_2 concentrations. Associations are resented for the following increments in exposure: 10 µg/m³ for NO_2, 20 µg/m³ for NO_x, 1 unit for $PM_{2.5}$ absorbance, 5 µg/m³ for $PM_{2.5}$, 10 µg/m³ for PM_{10}, 5 µg/m³ for PM_{coarse}, 5,000 veh·day^{-1}·m for traffic intensity on the nearest street; and 4,000 veh-km/day ·m for traffic load on major roads within a 100 m buffer.
